# Modeling How Heterogeneity in Cell Cycle Length Affects Cancer Cell Growth Dynamics in Response to Treatment

**DOI:** 10.3389/fonc.2020.01552

**Published:** 2020-09-10

**Authors:** Eleftheria Tzamali, Georgios Tzedakis, Vangelis Sakkalis

**Affiliations:** Computational Bio-Medicine Laboratory, Institute of Computer Science, Foundation for Research and Technology-Hellas, Heraklion, Greece

**Keywords:** cell cycle variation, intrinsic heterogeneity, cytotoxicity, phenotypical selection, cellular automata, mathematical modeling, resistance, recurrence dynamics

## Abstract

Tumors are complex, dynamic, and adaptive biological systems characterized by high heterogeneity at genetic, epigenetic, phenotypic, as well as tissue microenvironmental level. In this work, utilizing cellular automata methods, we focus on intrinsic heterogeneity with respect to cell cycle duration and explore whether and to what extent this heterogeneity affects cancer cell growth dynamics when cytotoxic treatment is applied. We assume that treatment acts on cancer cells specifically during mitosis and compare it with a (cell cycle-non-specific) cytotoxic treatment that acts randomly regardless of the cell cycle phase. We simulate the spatiotemporal evolution of tumor cells with different initial spatial configurations and different cell length probability distributions. We observed that in heterogeneous populations, strong selection forces act on cancer cells favoring the faster cells, when the death rates are lower than the proliferation rates. However, at higher mitotic death rates, selection of the slower proliferative cells is favored, leading to slower post-treatment regrowth rates, as compared to untreated growth. Of note, random cell death progressively eliminates the slower proliferative cells, consistently, favoring highly proliferative phenotypes. Interestingly, compared to the monoclonal populations that exhibit complete response at high random death rates, emergent resistance arises naturally in heterogeneous populations during treatment. As divergent selection forces may act on a heterogeneous cancer cell population, we argue that treatment plan selection can considerably alter the post-treatment tumor dynamics, cell survival, and emergence of resistance, proving its significant biological and therapeutic impact.

## Introduction

One of the major reasons for therapy failure in several cancer types has been attributed to the extensive heterogeneity observed among cells of a single tumor (intra-tumoral heterogeneity) and between different patients (inter-tumoral heterogeneity). Both intrinsic (i.e., genetic) and extrinsic (i.e., microenvironmental) factors may contribute to the heterogeneity observed, which significantly affects tumor progression and therapeutic effectiveness. The observed heterogeneity can be *spatial*, where genetically diverse subpopulations dominate spatially distinct tumor regions, or *temporal*, where the genetic makeup evolves over time in response to different types of selective pressures, including therapeutic intervention (1–8).

Cancer cells are characterized by dysregulated mitosis that boosts proliferation ability as compared to normal cells, leading to aggressive tumor progression (9). Several types of cytotoxic drug compounds are used in cancer therapy. Most of these compounds target protein components expressed during mitosis with the aim to block cell division and subsequently to induce cell death. The cytotoxic compounds that act during a specific phase of the cell cycle are known as cell cycle-specific drugs (such as paclitaxel and methotrexate), whereas those acting regardless of the cell cycle phase, targeting at any point in the cell cycle including the resting phase, are called cell cycle-non-specific agents (such as alkylating drugs and platinum analogs like cisplatin) (10). Available therapies are successful in some cancer types; yet, the extensive heterogeneity observed in many cancers turns therapeutic agents ineffective, promoting tumor recurrence and resistance.

Although many proposed mathematical models account for the natural variability in the proliferative capacity of the cell population by introducing heterogeneity in the cell cycle duration (11–14), the direct effect this heterogeneity plays on tumor evolution, when cytotoxic treatment is applied, has not been demonstrated. Differences between mitotic and random death rates reflecting different drug pharmacodynamics have not been demonstrated either. In particular, Powathil et al. (13) developed a hybrid discrete-continuous multi-scale mathematical model in order to study how spatial heterogeneity in oxygen that develops as tumors grow affects the efficacy of cell cycle phase-specific cytotoxic drugs. Greene et al. (14) and Lorz et al. (12) proposed an age-structured compartmental model that accounts for cell intrinsic heterogeneity in proliferation and apoptosis. In their work, they investigated how antimitotic drugs that primarily extend the cell cycle length and then trigger apoptosis affect cancer growth dynamics. Gallaher et al. (11) proposed a lattice-free agent-based model in order to explore how a heterogeneous distribution of phenotypes evolves over time. In their work, they accounted for both cell proliferation and migration, while they assumed a resistance cost to proliferating cells, such that phenotypes with faster proliferating rates are highly sensitive to the drug, whereas phenotypes with slower proliferating rates are more resistant. They concluded that different tumors require different treatments and reported that a continuous treatment strategy is better for homogeneous tumors, whereas adaptive therapy performs better in heterogeneous cancer populations.

In this work, we aim to investigate whether and to what extent intrinsic heterogeneity, with respect to cell cycle duration, affects cancer cell growth dynamics when cytotoxic treatment is applied. A cellular automaton model is used, assuming that each cancer cell lies on a two-dimensional (2D) regular lattice and is distinguished by its phenotypic properties. We assume that the tumor population is heterogeneous consisting of cells with different proliferation rates. Our findings are further compared against a homogeneous population to highlight the different behavior. Our aim is to build a simple, but biologically meaningful, model that allows us to focus on this form of heterogeneity and explore its potential impact on tumor progression before, during, and after treatment. Similarly to Gallaher et al. (11), the cells are initially seeded with two different configurations—one randomly scattered that mimics 2D *in vitro* experiments and another highly compact that mimics a central plane of a 3D tumor. We also assume that during treatment, cancer cells may die with a given probability that can be associated with the dose of an anticancer drug. This probability is either applied at the exact time a proliferating cell undergoes mitosis or randomly applied any time during the cell life. Although many experimental works (11, 15) report that drug-resistant cancer cells are, in general, less proliferative than drug-sensitive cells and that probably such a different sensitivity exists *a priori* in cells (before their exposure to treatment), in our work, we assume that all cells are equally sensitive/resistant to treatment. The rationale behind this assumption is to explore whether such a sensitivity/resistance may naturally emerge in the population. We investigate the spatiotemporal evolution of cells, as well as the evolution of the distribution of their proliferation times, as we vary the probability of a cell to die, imposing either mitotic or random death. We study these evolutions under different therapeutic schemes. Divergent selection forces acting on the heterogeneous cancer cell population and the emergence of resistant phenotypes are interestingly revealed.

## Materials and Methods

### Cellular Automaton Model

We assume that tumor cells lie on a 2D regular lattice. Each lattice site (20 × 20 μm) can accommodate only one tumor cell. A similar mathematical description has been presented (16–18). The cells are seeded with two different initial configurations—one circular but randomly scattered of low cell density (1%) that mimics 2D *in vitro* experiments and another circular but highly compact (80%) that mimics a central 2D plane of a dense 3D tumor. In the first configuration, an initial population of 5,000 cells is sparsely scattered throughout a circular area of 8 mm radius. In the second configuration, we initially seeded 1,000 cells, tightly placed in a 0.4 mm radius area. We assume that the tumor population is heterogeneous consisting of cells with different proliferation rates. In this work, this property is intrinsic, inherited, and microenvironmental-independent and thus does not change throughout our experiments. In order to study whether our conclusions depend on differences in the initial distribution of cells, we also assume two different initial distributions for the doubling times; normal and uniform with the same mean τ and variance τ/5. We assume τ equals to 24 h. We started with 500 phenotypes randomly drawn from these distributions. Thus, 500 phenotypes are randomly drawn from either the normal distribution *N[*τ, √*(*τ*/5)]* or the uniform distribution *U(15.7 h, 32.3 h)*. A homogeneous population in which all cells have the same doubling time equal to τ is also explored for comparison.

For simplicity, cell motility is not taken into account. Tumor cells can be found in one of the following states—actively dividing, quiescent due to space competition, or dead as the result of treatment. We assume that when a cell dies, lysis is rapid and an empty space is instantly created in its place. At the beginning of the simulations, each tumor cell is randomly assigned an age, which corresponds to the time spent in the cell cycle and increases at each update of the model until the cell completes mitosis and is divided. Division is possible when available free space is found in the 2-Moore neighborhood; otherwise, the cell becomes quiescent. The divided cells reset their age. To avoid possible synchronization artifacts during division, particularly in the homogeneous populations, a small zero mean noise term is added to the age increments.

### Treatment

During treatment, cancer cells may die with a given probability that in general can be associated with the dose of an anticancer drug. In one scenario, this probability is applied at the exact time a proliferating cell undergoes mitosis, noted as mitotic death probability, *p*_*m*_. In the alternative scenario, the probability is applied randomly in any time during the cell cycle, even if the cell is quiescent, noted as random death probability, *p*_*r*_. To achieve the same death rate, λ for mitotic and random death probabilities, we describe random death probability assuming a Poisson probability distribution, where the death rate λ reflects the probability (*dp*_*r*_) per unit time (*dt*) that a cell will die. Thus, *dp*_*r*_ = λ*dt*, where λ = −μ*p*_*m*_ and μ = ln 2/τ.

We explore different therapeutic schemes in order to understand how heterogeneity and the cancer population evolve during treatment, as well as after treatment. In particular, we investigate the impact of (i) long, continuous treatment that lasts throughout the whole experiment; (ii) switch-on/switch-off treatment, where treatment is applied for a relatively short period of time and then is ceased for the rest of the experiment; and (iii) periodic switch-on/switch-off treatment.

## Results

We investigate the spatiotemporal evolution of cells and the evolution of the distribution of their proliferation times, as we vary the probability of a cell to die. Differences between homogeneous and heterogeneous populations are explored, as well as differences between mitotic and random death probabilities. Each experiment has been repeated five times in both low and highly dense initial configurations. Firstly, we present the results where an initially low cell density is assumed in both untreated and constantly treated settings. In these experiments, we have chosen to present the mean and variance of doubling times from a single experiment in order to highlight the intra-tumoral heterogeneity. The mean and variance across the multiple experiments (inter-experiment consistency) can be found in the Figures S1, S2, S6. Then, we present the results of the highly dense initial configuration. In these examples, we present the mean and variance across the experiments. Apart from constant treatment, switch-on/switch-off treatment, as well as periodic switch-on/switch-off treatment, is explored. The simulations run for 1,200 and 2,400 h, respectively, for the sparse and dense configurations, unless a cell reaches the edge of the computational domain L within proximity of L/10 cells.

### Space Competition and Selection in Untreated Population

**Figure 1:**
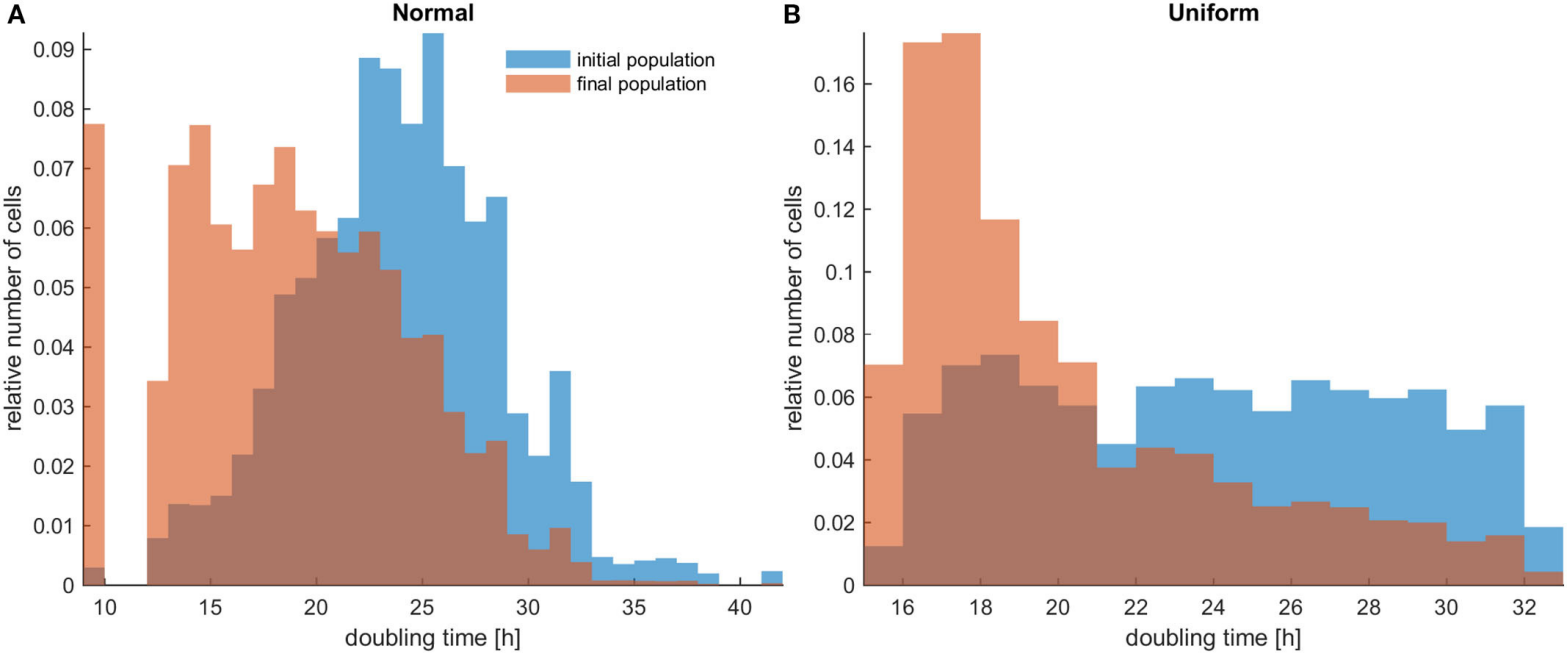
The distributions of doubling times within a population at the beginning and at the end of the experiments for untreated heterogeneous cells in low initial cell density. The simulations illustrated are from a single experiment. **(A)** Initially normal distribution (depicted with blue color) and how it evolves (depicted with orange/salmon color). **(B)** Initially uniform distribution (depicted with blue color) and how it evolves (depicted with orange/salmon color). As time progresses, both distributions have evidently become right-skewed.

**Figure 2:**
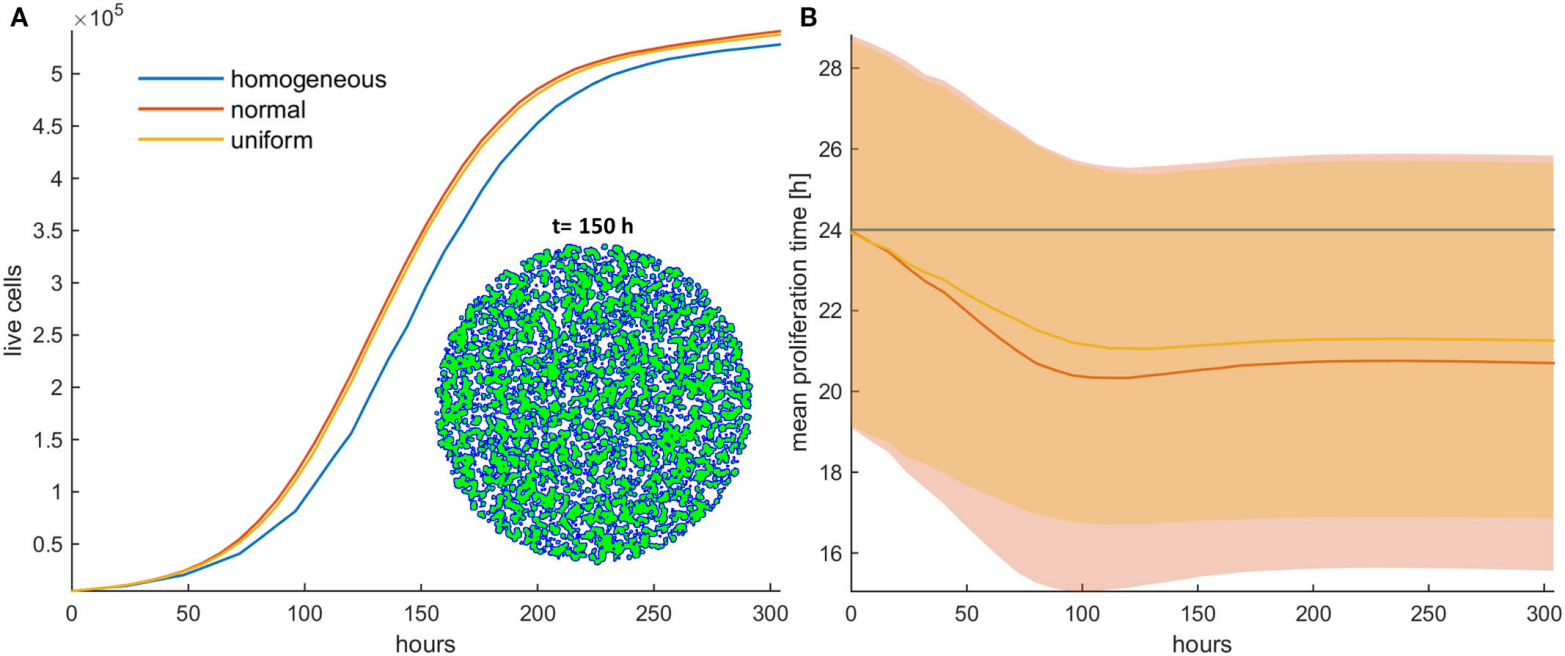
**(A)** Growth curves and **(B)** the evolution of the mean doubling time (mean and standard deviation values) of an untreated cancer cell population in low initial cell density for the homogeneous (blue color), the initially normal (red color), and the uniform (yellow) distributions. The spatial distribution of cells is also shown at *t* = 150 h for the initially normal distribution of phenotypes. In this snapshot, proliferating cells are illustrated with blue color and quiescent cells are green. The plateau that the mean proliferation time reaches after some time is the result of increased quiescence in the population that hinders the selection process. The simulations presented are from a single experiment.

Firstly, the evolution of an untreated population is explored in an initial population of low cell density. As expected, phenotypes with proliferative advantage grow rapidly. We observe that as time progresses, the faster phenotypes dominate. In particular, we observe that the distributions of doubling times are becoming right-skewed for both the initially normal (Figure 1A) and uniform distributions (Figure 1B). Consequently, the mean doubling time is reduced as the system evolves (Figure 2B). The consequence of this selection is also reflected in the growth curves where the heterogeneous populations show slighter increases relative to the homogeneous one (Figure 2A). Note that although both distributions have initially the same variance, the range of their values differs. We also observe that the plateau the mean proliferation time reaches after some time is the result of increased quiescence in the population. The onset of quiescence starts at ~*t* = 100 h, and after that, the population becomes progressively compact with increased number of quiescent cells. A snapshot of the cell distribution at *t* = 150 h is also illustrated in Figure 2A. The proliferating cells are colored blue. The initially sparse cell distribution with a lot of empty space in between (depicted with white color) becomes significantly compact with an increased number of quiescent cells (depicted with green color). Thus, space competition hinders phenotypic selection, a phenomenon that becomes particularly evident in untreated populations where space competition becomes the only limiting factor of growth.

### Selection Forces Depend on Mitotic Death Probability

**Figure 3:**
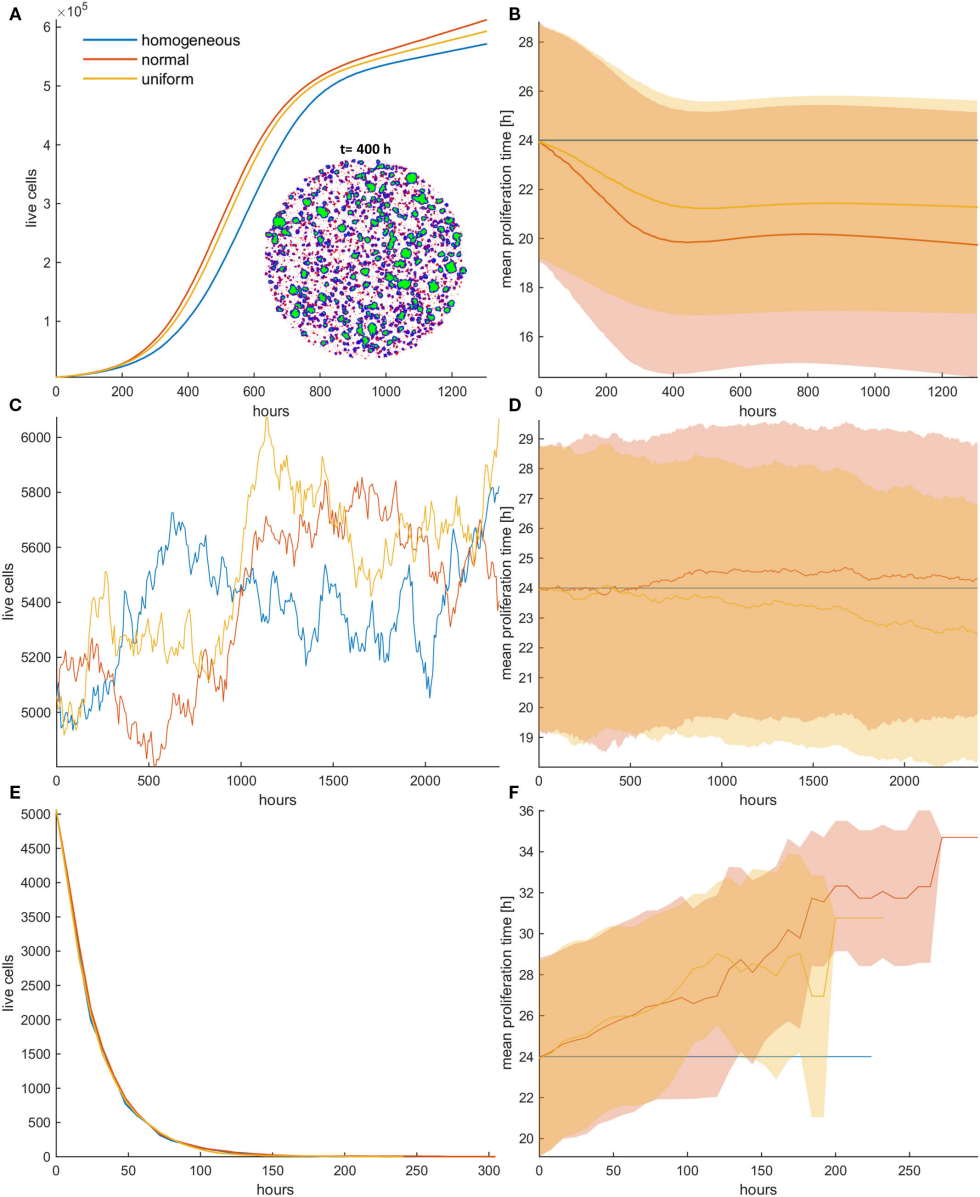
**(A,C,E)** Growth curves and **(B,D,F)** the evolution of the mean doubling time (mean and standard deviation values) of a treated cancer cell population with **(A,B)** low mitotic death probability (*p*_*m*_ = 0.4), **(C,D)** mitotic death probability equal to 0.5, and **(E,F)** high mitotic death rate (*p*_*m*_ = 0.8). The simulations illustrated are from a single experiment. Low initial cell density is assumed for the population. The evolution of the homogeneous (blue color), the initially normal (red color), and the uniform (yellow) distributions is shown for each case. The spatial distribution of cells at *t* = 400 h is also illustrated in panel **(A)** for the initially normal distribution, when *p*_*m*_ = 0.4. In the embedded snapshot, proliferating cells are shown in blue, quiescent are green, and necrotic cells are red. Necrotic cells are uniformly distributed within the population, yet they cannot be found within the quiescent islands. Compared to the untreated population, the mitotic death delays the selection process. As the death rate equals the proliferation rate **(C,D)**, the population remains practically constant over time **(C)**. Furthermore, it is evident that there is no selection force to favor phenotypes with either slower or faster proliferation rates **(D)**. At high death rates **(E,F)**, the growth curve **(E)** is dominated by the increased death rate and declines over time similarly for all the distributions; yet, slower phenotypes with increased doubling time **(F)** are now progressively selected.

Next, we investigate the role of applying mitotic death probability in an initial population configuration of low cell density. We assume constant treatment, applied throughout the whole experiment. As shown in Figures 3A,B, similarly with the untreated population, we observe that the faster phenotypes dominate in the heterogeneous populations with low mitotic death probabilities (*p*_*m*_ < 0.5). Figures 3A,B show the growth curves for the homogeneous (depicted with blue color), the initially normal (depicted with red color), and the initially uniform distribution (depicted with yellow color) of phenotypes. A snapshot showing the cell distribution at *t* = 400 h for the initially normal distribution of phenotypes is also illustrated in Figure 3A. This is approximately the time where the onset of quiescence starts, which is 4-fold longer compared to the untreated population. At this time, the mean proliferation time reaches a plateau (Figure 3B). Thus, compared to the untreated populations, the mitotic death delays the selection process, although both cases eventually reach approximately the same plateau value. The spatiotemporal evolution of the tumor population indicatively for the initially normal distribution of phenotypes can be seen in the Video SV1 for mitotic death probability equal to 0.4. When the inner region of the tumor population becomes quiescent, the selection of phenotypes in that region freezes, trapping the evolved phenotypes. Selection is then driven solely from the outer region.

As the mitotic death probability increases, the phenotypic selection process progressively delays. When the mitotic death probability equals to the growth probability (*p*_*m*_ = 0.5), the growth curve of the heterogeneous cell population, as well as the mean doubling time, remains practically constant over time (Figures 3C,D), indicating that there is no selection force to favor phenotypes with either slower or faster proliferation rates in this case. On the other hand, we interestingly observe that for high mitotic death probabilities (*p*_*m*_ > 0.5), in which the death rate is larger than the proliferation rate, the mean doubling time increases over time (Figures 3E,F), indicating that phenotypes with slower proliferation rates are now selected. The growth curve is dominated by the increased death rate and declines over time similarly for all the distributions (Figure 3E). The distributions of doubling times at the end of the simulations are illustrated in the Figure S3 for all cases. Overall, we conclude that depending on the mitotic death probability, different selection forces act on phenotypes, which can either favor faster or slower phenotypes.

### Random Death Probability During Constant Treatment Always Favors Faster Proliferating Cells and Allows the Emergence of Resistance

**Figure 4:**
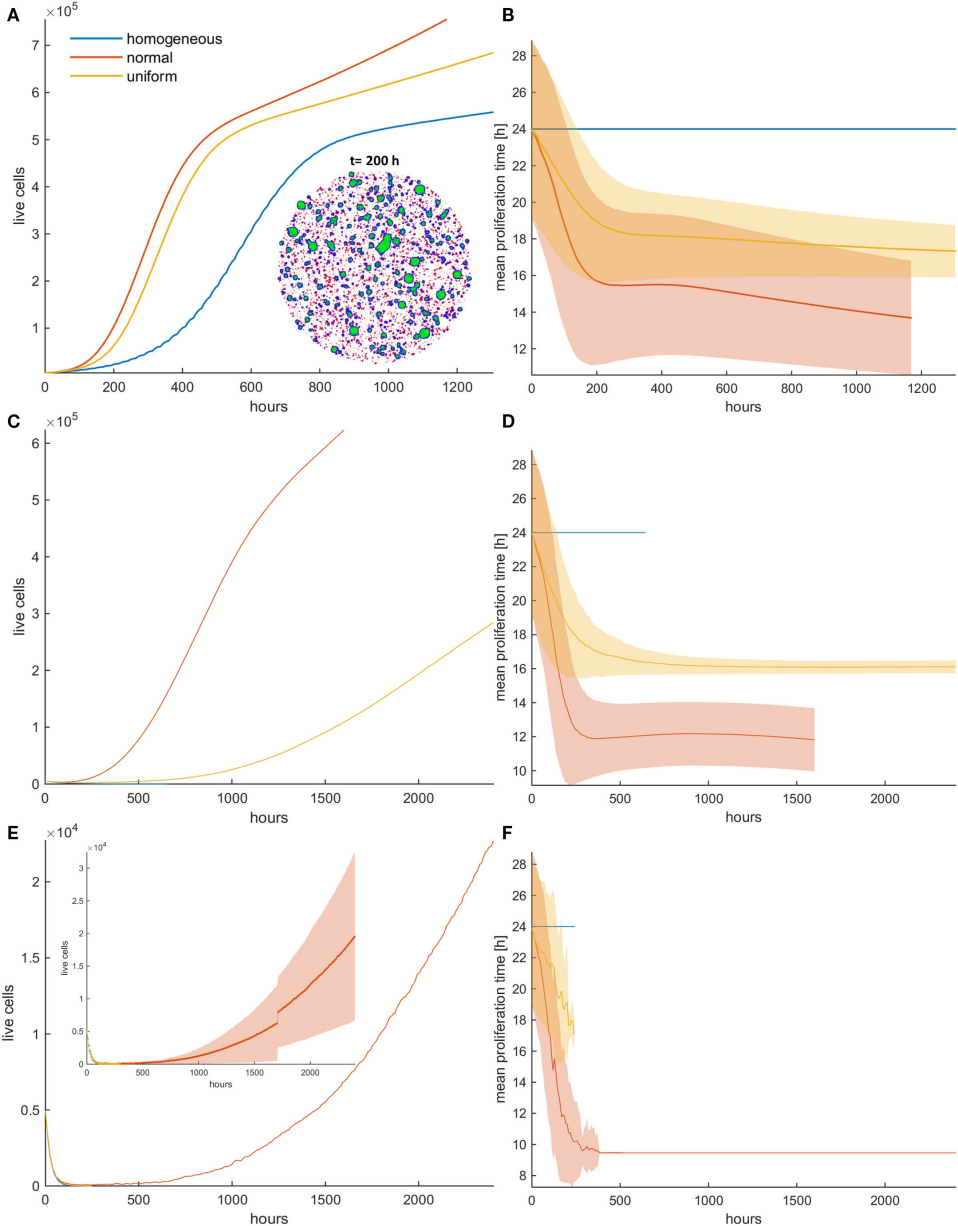
**(A,C,E)** Growth curves and **(B,D,F)** the evolution of mean doubling time (mean and standard deviation values) of a treated cancer population in low initial cell density for **(A,B)** low (*p*_*r*_ = 0.4), **(C,D)** middle (*p*_*r*_ = 0.6), and **(E,F)** high (*p*_*r*_ = 0.8) random death rates. The simulations illustrated are from a single experiment. The spatial distribution of cells at *t* = 200 h is also illustrated in panel **(A)** for the initially normal distribution, when *p*_*r*_ = 0.4. In the embedded snapshot, proliferating cells are shown in blue, quiescent are green, and necrotic cells are red. Necrotic cells are uniformly distributed within the population and can be found even within the quiescent islands. Selection consistently favors highly proliferating cells with considerable impact on growth response dynamics. Differential response period to treatment and population recurrence is particularly evident when middle and high random death rates are applied. The embedded figure in panel **(E)** shows the mean and standard deviation of the growth curve across five experiments. After a long response period, recurrence is highly probable in the heterogeneous populations when initialized with normal distribution.

We now investigate the role of applying random death probability in an initial population configuration of low cell density. Figure 4 shows the growth curves (Figures 4A,C,E) and the evolution of the mean doubling time (Figures 4B,D,F) for the homogeneous and the heterogeneous population when the random death probability is relatively low (*p*_*r*_ = 0.4; Figures 4A,B), middle (*p*_*r*_ = 0.6; Figures 4C,D), and high (*p*_*r*_ = 0.8; Figures 4E,F). Contrary to mitotic death probability, we observe that faster phenotypes are systematically selected at either low or high random death probabilities. The rationale behind these observations is that when random death probability is applied, all cells in a population experience a fixed death rate that corresponds to this probability, regardless of their proliferation rate. This means that slower proliferating cells will be affected the most by this probability. On the contrary, in the mitotic death probability, the death rate adapts to proliferation rate. Thus, while growth and mitotic death rates drive selection in opposite directions, random death rates select phenotypes in the same direction with growth. Furthermore, as random death can also affect quiescent cells, space competition is reduced. This, together with the consistent filtering out of the slower phenotypes, accelerates the selection process of the faster phenotypes, resulting in dramatic differences in the growth dynamics between the homogeneous and heterogeneous populations. As can be seen in Figure 4, the selected phenotypes drive the evolution of the population showing a differential response period to treatment and population recurrence. For example, when *p*_*r*_ = 0.6 (Figures 4C,D), we can observe substantial differences in the growth curves between the homogeneous and the heterogeneous populations, as well as between normal and uniform distributions. Because of the wider range of values that the phenotypes can possess in the normal relative to uniform distribution, very fast phenotypes are selected, leading the cancer population to rapid recurrence. Note that the homogeneous population responds to treatment throughout the whole experiment. The increased sensitivity of the homogeneous populations becomes evident without any *a priori* assumption related to treatment response. In addition, we also interestingly observed that at high random death rates (Figures 4E,F), the initially normal distributed phenotypes possess the heterogeneity necessary, so that resistance emerges naturally after a long period of response, where only very few cells have managed to survive. From these few highly proliferative cells, the population is capable of regrowing. In order to demonstrate the consistency of this observation, the embedded figure in Figure 4E shows the growth curves (mean and standard deviation values) for each distribution across five experiments. As can be seen, recurrence is highly probable in the heterogeneous populations when initialized with normal distribution. Note that other mechanisms, like the Allee effect (19, 20), might play a critical role at these extremely low densities of recurrence, probably prohibiting the growth of these populations in real settings. The spatiotemporal evolution of the tumor population indicatively for the initially normal distribution of phenotypes can be seen in the Video SV2 for random death probability equal to 0.4. Contrary to mitotic death probability, slower proliferating cells are not permanently trapped to quiescent regions. As random death does not affect only the proliferating cells, there is constant selection of phenotypes, even when the inner region of the tumor population becomes quiescent. Furthermore, the distributions of doubling times at the end of the simulations are illustrated in the Figure S7 for all cases.

### Spatial Selection in Dense Configurations Under Constant Treatment

**Figure 5:**
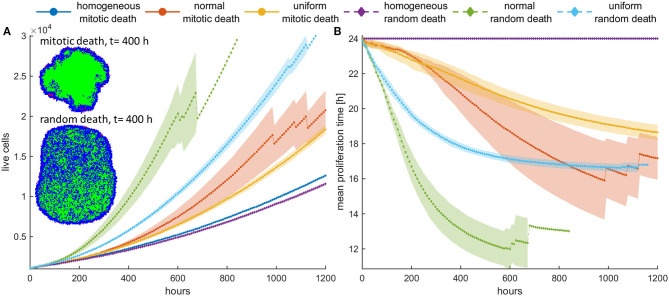
**(A)** Mean growth curves and **(B)** the evolution of the mean doubling time across five experiments of a treated cancer cell population in high initial cell density when either mitotic (*p*_*m*_ = 0.4) or random (*p*_*r*_ = 0.4) death probability is applied. The standard deviation values across the five experiments are also shown. The cancer cell population grows much faster when random death is applied as compared to the mitotic death because the underlying selection forces act in the former case on the same direction. The spatial distribution of cells at *t* = 400 h is illustrated in panel **(A)** for the initially normal distribution of phenotypes. In this snapshot, proliferating cells are illustrated with blue color, quiescent cells are green, and necrotic cells are depicted with red color. Dramatic difference in the expansion dynamics of the tumor between mitotic and random death is apparent. When random death is applied, necrotic cells are homogeneously dispersed within the tumor. However, when mitotic death is applied, the necrotic cells appear only within the proliferating rim where mitosis occurs.

We now consider the effect of random and mitotic death probabilities in highly dense initial configurations that resemble a dense tumor mass. Figure 5 shows the evolution of the mean growth curves (Figure 5A) and the evolution of the mean doubling time (Figure 5B) across five experiments for low mitotic and random death probabilities. Even though the space competition is now significantly higher relative to the low dense configuration, and the population expansion comes solely from the tumor rim, similar behaviors are observed. In particular, we observe the selection of faster phenotypes at low mitotic death rates and untreated conditions, as well as the selection of slower phenotypes at higher mitotic death rates (Figure 5, Figures S4, S5). When random death is applied, selection of faster phenotypes is consistently observed (Figure 5, Figure S8). Similarly to the low dense configurations, considerable differences are observed in the growth dynamics between random and mitotic death probabilities, as well as between homogeneous and heterogeneous populations, which are explained by the variation in the selection forces that act on the population.

However, we should note that in dense configuration, the results of the divergent selection forces between random and mitotic death probabilities have an additionally dramatic effect in the expansion dynamics of the tumor. Figure 5A shows the spatial distribution of a cancer cell population at *t* = 400 h, when (a) mitotic and (b) random death probability is applied (*p*_*m*_ = *p*_*r*_ = 0.4). The proliferating cells are shown with blue color, the quiescent cells are green, and the necrotic cells are colored red. It is evident how the consistent selection of fast proliferating phenotypes results in tumors with increased sizes. Furthermore, we can observe that when mitotic death is applied, the quiescent region is more homogeneously distributed in the inner region of the tumor mass, whereas the necrotic cells appear within the proliferating rim where mitosis occurs. On the other hand, when random death is applied, necrotic cells are homogeneously dispersed within the tumor, as random death acts equally in both proliferating and quiescent cells. A significant number of proliferating cells can also be found scattered within the tumor mass, taking over the place of the necrotic cells.

**Figure 6:**
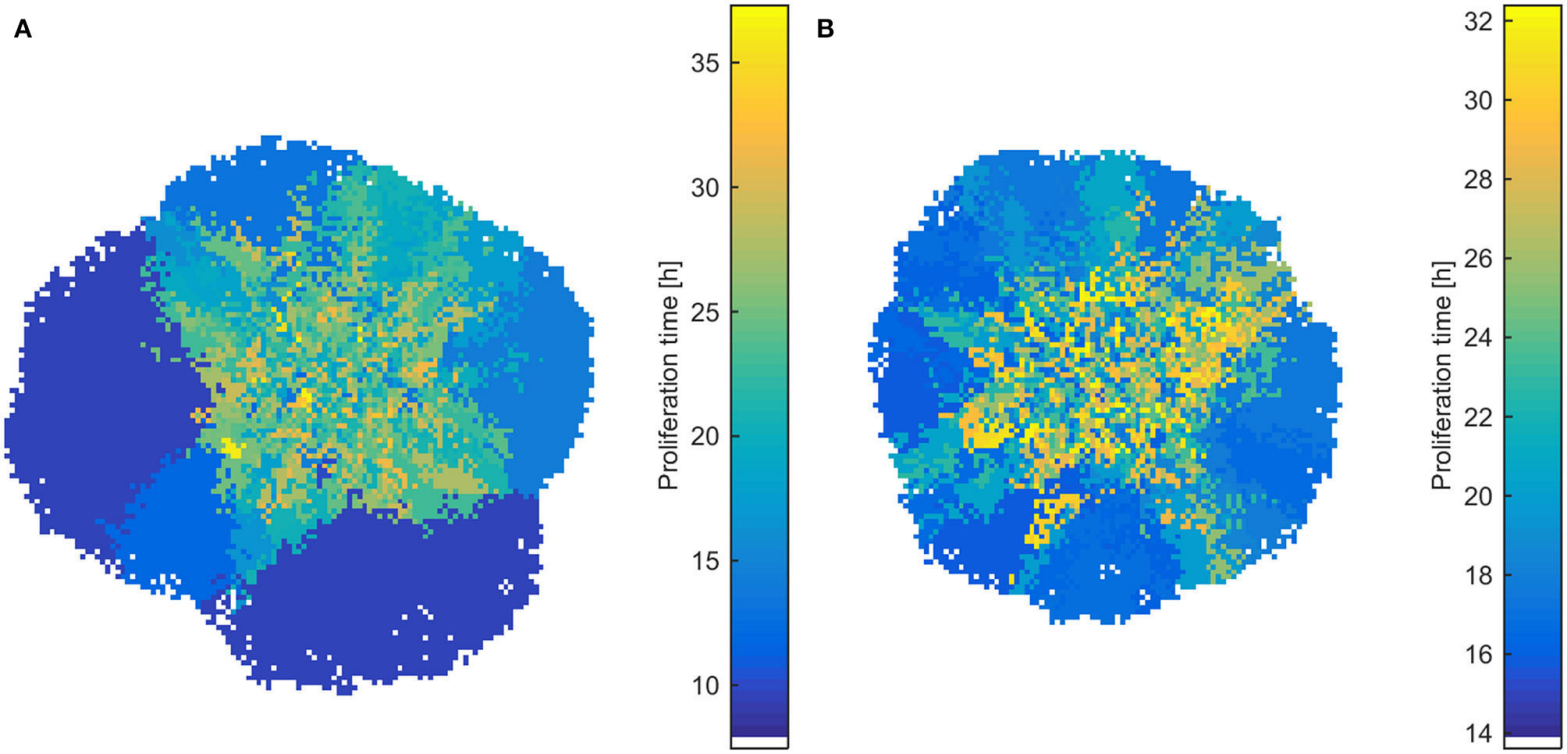
Snapshots of heterogeneous dense tumors at *t* = 600 h showing the spatial distribution of phenotypes for mitotic death probability equal to 0.4. **(A)** Example of an initially normal and **(B)** uniform distribution of phenotypes is shown. The color bar shows the corresponding doubling time (in h) of the phenotypes. Bluish phenotypes are more proliferative, whereas yellowish phenotypes have longer doubling times. Slower proliferating cells are trapped to inner regions, while highly proliferative phenotypes take over the expanding front.

Interestingly, the selective process also affects the spatial distribution of phenotypes, as well as the shape of the evolved tumor, which deviates from the rounded shape of the homogeneous tumors. As can be seen in Figure 6, slower proliferating cells are trapped to inner regions, while highly proliferative phenotypes take over the expanding front, similarly to what has been observed before (11, 21). These highly proliferating cells also drive the growth dynamics of the tumor. The effect is even more pronounced in heterogeneous populations initialized with normal distribution, where the different range of possible proliferation times allows extremely fast phenotypes to eventually dominate and change the shape and expansion rate of the tumor, even if initially rare in the population. In support of other works, multiple sampling from spatially distinct tumor regions must thus be preferred, contrary to the most common single tissue biopsy, especially for highly heterogeneous tumors (6, 21). The spatiotemporal evolution of the tumor population for the initially normal distribution of phenotypes can be seen in the Videos SV3, SV4 for mitotic and random death probability equal to 0.4.

### Divergent Selection Forces Determine Post-treatment Dynamics: Switch-On/Switch-Off Treatment in Dense Configuration

**Figure 7:**
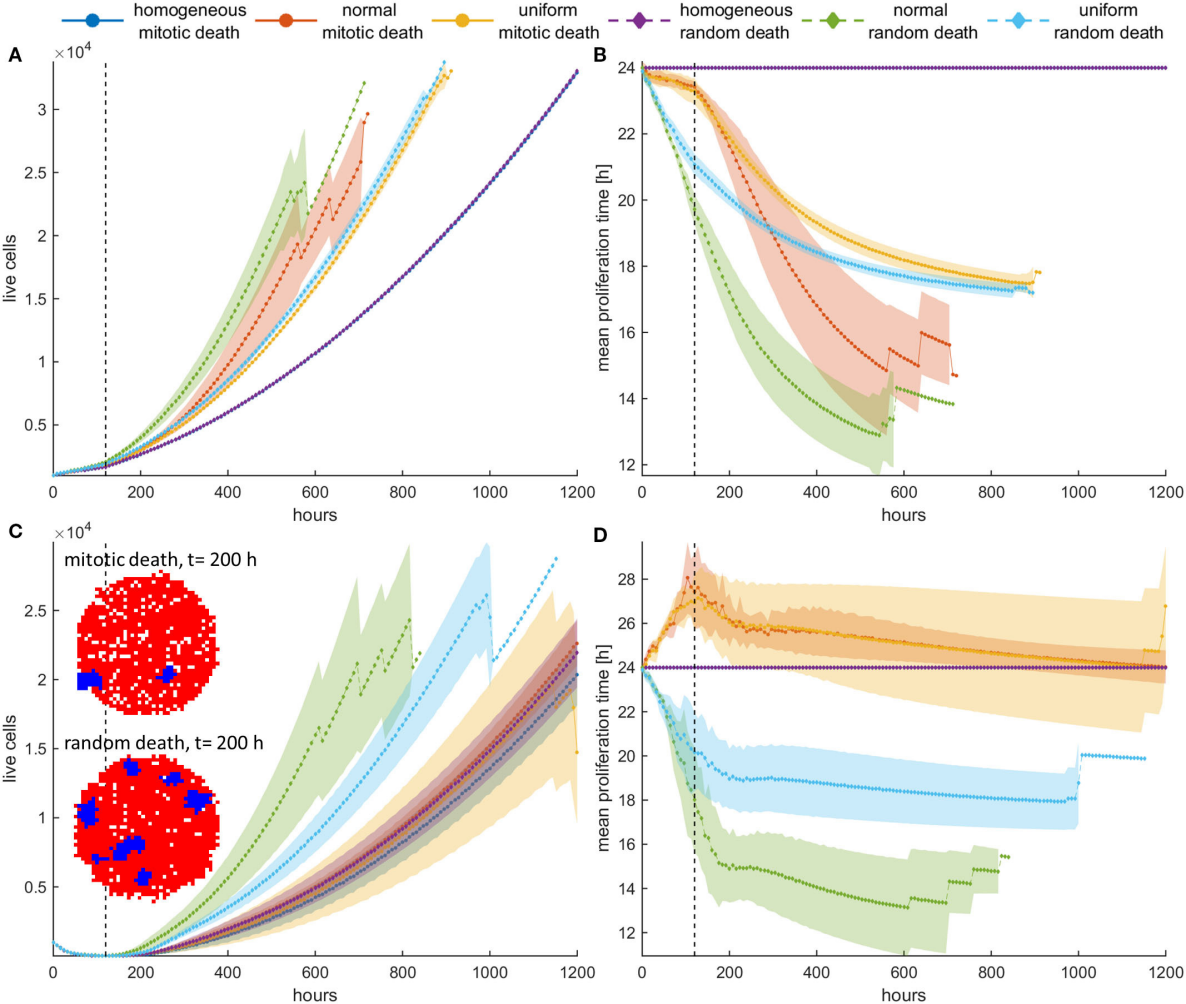
**(A,C)** Mean growth curves and **(B,D)** the evolution of the mean doubling time of a treated cancer population in dense configurations for **(A,B)** *p*_*m*_ = *p*_*r*_ = 0.4 and **(C,D)** *p*_*m*_ = *p*_*r*_ = 0.8 applied the first 120 h (5 days). The vertical dotted line dictates when treatment stops. After that period, the population is left untreated. The experiments have been repeated five times. The mean and standard deviation across the five experiments are shown for each case. The divergent selection forces during and after treatment are evident. The spatial distribution of cells for the homogeneous tumors near the time recurrence is visible (*t* = 200 h) is also shown. In this snapshot, proliferating cells are illustrated with blue color and necrotic cells are depicted with red color. Just after the dispersed foci of surviving cells are reunited to form a new tumor, the growth dynamics of the homogeneous tumors coincide.

**Figure 8:**
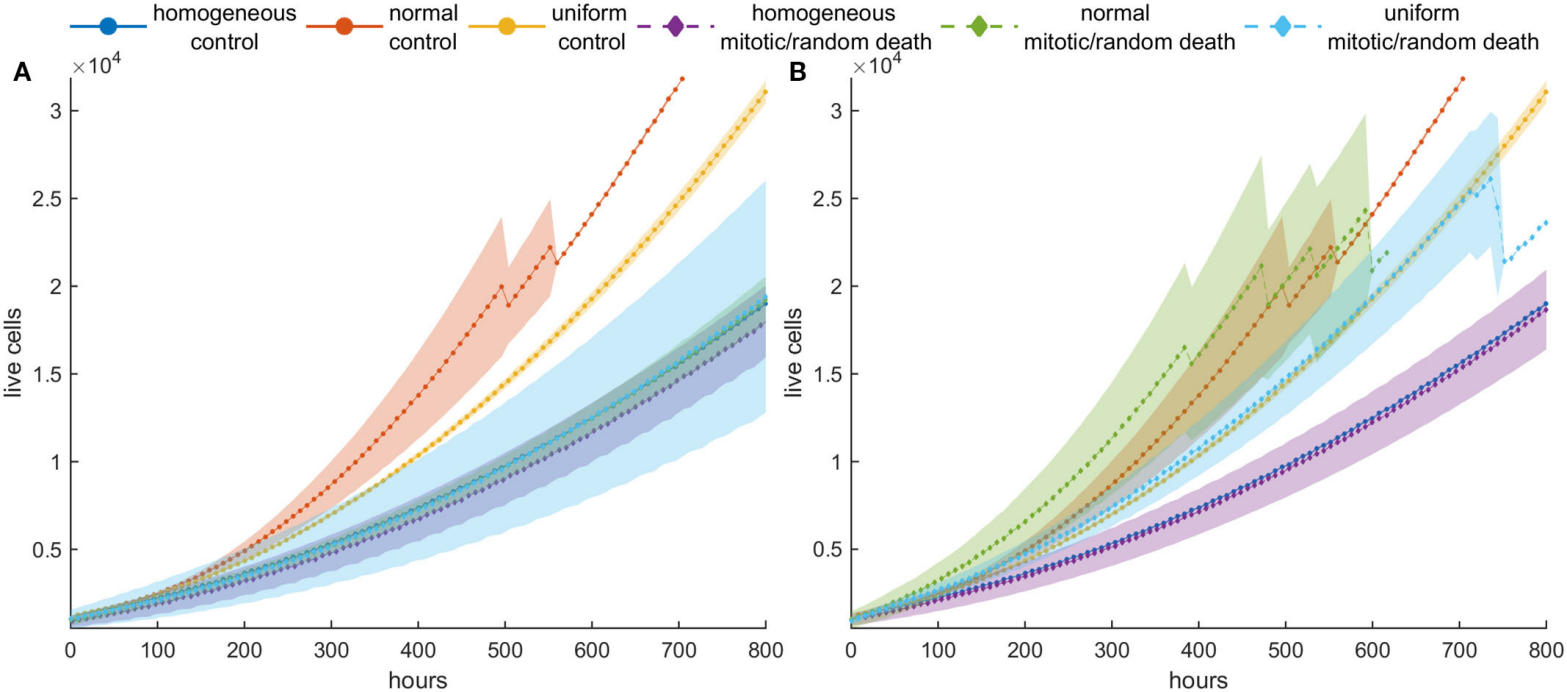
Mean post-treatment growth curves compared with the corresponding untreated (control) case in dense configurations. Treatment at high death probability (*p*_*m*_ = *p*_*r*_ = 0.8) has been applied for 5 days. **(A)** Recurrence dynamics are slower relative to untreated growth dynamics when high mitotic probability is applied. **(B)** Recurrence dynamics are similar or slightly faster relative to untreated growth dynamics when a high random probability is applied.

To further highlight the impact the previous observations have upon recurrence, we now try a different therapeutic scheme where we apply treatment only the first 5 days and then we leave the population untreated. An initially highly dense configuration is assumed. Although the homogeneous populations have very similar dynamics before and after treatment, with no differences between random and mitotic death rates (Figures 7, 8), the heterogeneous populations evolve remarkably different. Note that the slight discrepancy in the recurrence growth curve between mitotic and random death of the homogeneous population at high death rates (Figure 7C) is due to the fact that the population regrowth initiates from different starting points with respect to population size, as illustrated in the snapshots presented in Figure 7C. Curve fitting of the growth dynamics shows that after that reinitialization period, where the foci of surviving cells are reunited to form a new tumor, the growth dynamics of the homogeneous tumors coincide (Figures S9, S10).

In general, we observe that for low mitotic death rate (Figures 7A,B), the post-treatment dynamics are faster in heterogeneous populations relative to homogeneous ones (Figure 7A), as faster phenotypes consistently dominate during and after treatment. We can also clearly observe that during treatment with mitotic death, selection is slower compared to after treatment dynamics, whereas for random death probabilities, the selection dynamics during and after treatment remain similar (Figure 7B).

At higher death rates (Figures 7C,D), we observe that when mitotic death rates are applied, the recurrence dynamics between the heterogeneous and the homogeneous population are very similar. This is because of the divergent selection forces that act on the tumor population during and after treatment. On the other hand, regrowth is faster when random death rates are applied compared to mitotic rates. Interestingly, however, when we compare the growth dynamics between the treated and the untreated population (control), we observe that the selection of slower phenotypes during treatment at high mitotic death rates results in slower recurrent dynamics (Figure 8)—an observation that has been seen in real tumors after antimitotic treatment (22). Note that this is not true for random death rates that always favor the highly proliferative phenotypes. Recurrence dynamics are very similar relative to untreated growth dynamics when high random probability is applied (Figure 8, Figure S9, S10).

### Periodic Switch-On/Switch-Off Treatment (Repeated Cycles)

We have previously seen that with one cycle of treatment, even at high death probabilities, population recurrence is inevitable for both homogeneous and heterogeneous cancer cell populations. We further investigate the effect of a periodic switch-on/switch-off treatment strategy on population dynamics, where treatment is applied for 5 days followed by 5 days of vacation, and then this cycle is repeated again and again multiple times. We applied this treatment strategy for five cycles in an initially dense population and explored how the population evolves.

**Figure 9:**
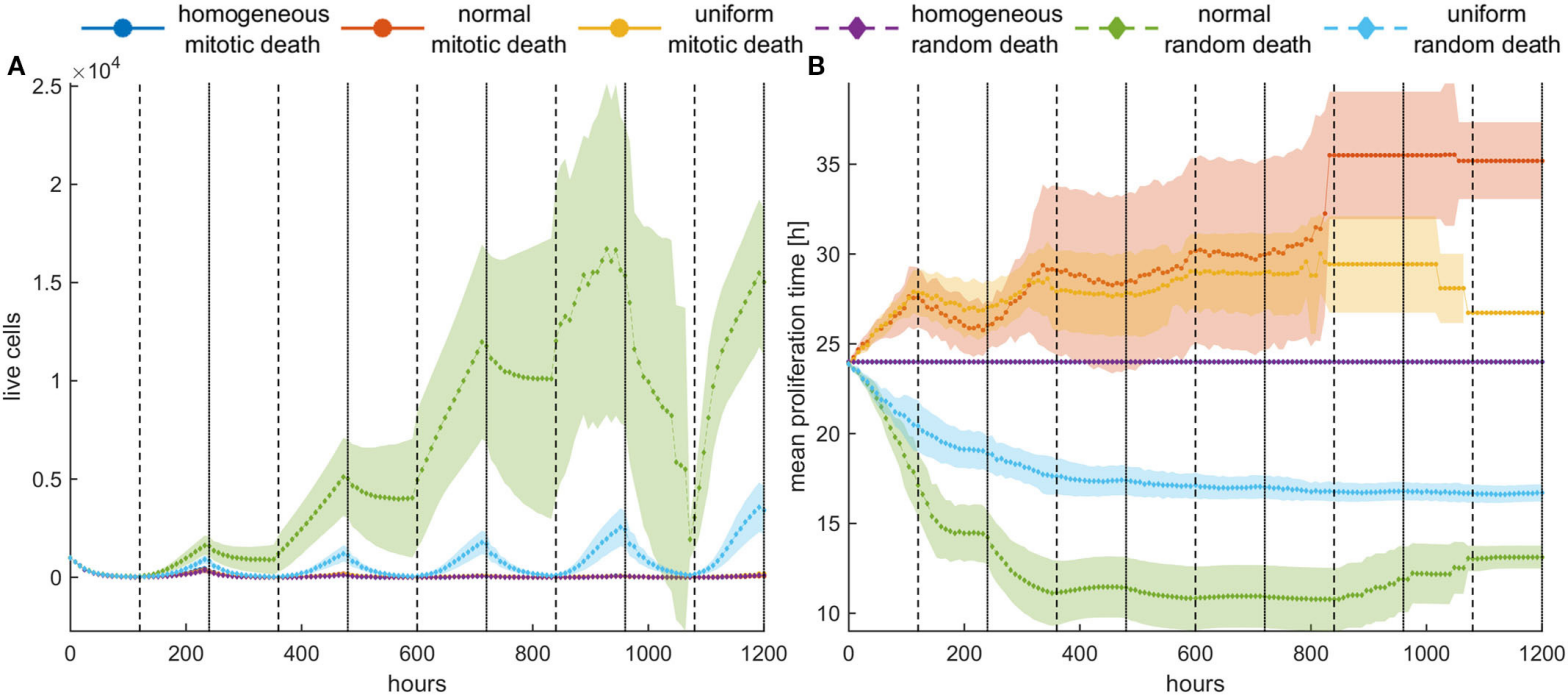
Periodic switch-on/switch-off treatment in dense configuration. Treatment is applied for 5 days (depicted with white) followed by 5 days of vacation (depicted with gray), and then this cycle is repeated five times. High death probabilities (*p*_*m*_ = *p*_*r*_ = 0.8) are applied during treatment. **(A)** The evolution of the cell population as well as **(B)** the evolution of the mean doubling time. The standard deviation across five experiments is also shown. Different underlying selection forces produce different therapeutic outcomes. Contrary to random death probabilities, homogeneous populations consistently remain under control, as well as heterogeneous populations under mitotic treatment.

Figure 9 shows the mean growth curves, as well as the evolution of the mean proliferation time for the heterogeneous and the homogeneous populations, when high death probabilities (*p*_*m*_ = *p*_*r*_ = 0.8) are periodically applied. We observe that when the death probability is applied at mitosis, tumor population remains small and stable over time contrary to random death probabilities that progressively increase their population. In the latter case, a different therapeutic scheme like adaptive therapy or schemes with shorter vacation periods could be more effective. On the other hand, the homogeneous populations consistently remain under control regardless of whether the probability is applied randomly or in mitosis. This is not surprising considering the underlying selection processes involved in each case. Yet, it is another interesting example demonstrating how the selected clones can determine the fate of therapy, making it either effective or ineffective. Therapy must be adapted depending on the selection processes.

## Discussion

Numerous mathematical models have accounted for heterogeneity in the tumor population (11–13, 22–25), as it affects various aspects of tumor evolution including growth, invasion, and therapy failure. Among them, many models have also accounted for the natural variability with respect to proliferation rate (11–13, 22). However, the direct effect this heterogeneity plays on tumor evolution without assuming any prior trade-off between fitness and drug sensitivity has not been demonstrated when cytotoxic treatment is applied. Even more, differences between therapy that acts specifically at mitosis and therapy acting regardless of the cell cycle have not been explored, to the best of our knowledge, for heterogeneous populations.

Utilizing a cellular automaton mathematical model, we investigate the spatiotemporal evolution of tumor cells and the evolution of the distribution of their proliferation times, as we vary the probability of a cell to die from a specific treatment. Differences between homogeneous and heterogeneous populations are explored, as well as differences between mitotic and random death probabilities. We also assume two different initial distributions for the doubling times; normal and uniform. Furthermore, the cells are initially seeded with two different configurations—one randomly scattered that mimics 2D *in vitro* experiments and another highly compact that mimics a central plane of a dense 3D tumor. Various therapeutic schemes have been also tested including constant, switch-on/switch-off, and periodic switch-on/switch-off treatments in order to demonstrate the importance of taking into account the underlying selection forces acting on a cell population in therapy.

We showed that as the mitotic death rate increases, the selection of faster phenotypes is inhibited, and eventually, slower phenotypes are favored at higher mitotic death rates. On the contrary, random death rates, regardless of whether they are greater or lower than the proliferation rates, always promote faster phenotypes. In that case, as highly proliferating cells escape, resistance emerges even at high random death rates and under constant treatment. Thus, without applying any prior trade-off, resistant phenotypes emerge and drive the evolution of the population, showing a differential response period to treatment and population recurrence. The underlying phenotypic selection forces are also reflected in the spatial distribution of phenotypes within a tumor, as well as on the shape of the evolved tumor. Slower proliferating cells are usually trapped to inner regions, while highly proliferative phenotypes take over the expanding front. Similar behaviors with competing phenotypes have been observed (11, 21). For example, in Gallaher et al. (11), sensitive cells, which are faster, and mutant subclones in Chkhaidze et al. (21) with fitness advantage are those taking over the outer, growing front, whereas resistant and less fit subclones are usually trapped within. However, we showed that at high mitotic death rates, where fast proliferating cells are highly affected and space competition is less evident, slower proliferating cells manage to dominate in the population. As selection forces rapidly act on the tumor population, the initial distribution of phenotypes also changes rapidly, deviating from the initially normal or random distribution. Differences between the two initial distributions are observed; yet, they are due to their different range/interval of doubling times.

The effect of the selection forces is becoming particularly evident in post-treatment recurrence dynamics. When therapy of high mitotic death rates ceases, we observe that the recurrence dynamics are slower relative to untreated populations, an observation that has been seen under antiproliferative treatments in glioblastoma (22). Yet, this is not true for random death probabilities that always favor faster proliferating phenotypes. Interestingly, we also observed that the homogeneous populations, as they lack heterogeneity, exhibit a complete response to increased random death rates, contrary to the polyclonal, heterogeneous populations where resistance emerges during treatment. Thus, homogeneous population can more easily remain under control. The importance of taking into account the underlying selection mechanisms, which are different between mitotic and random death, is also demonstrated when the periodic switch-on/switch-off treatment is applied. The simulations show that a heterogeneous population can more effortlessly remain under control when mitotic death is applied, but not when death occurs randomly in the population. In that case, it is important to provide adaptive therapy planning that readjusts according to the evolving proliferative capacity of the dominant phenotypes.

Overall, our simulations demonstrate that the selection forces acting on the heterogeneous populations may change the outcome far from the homogeneous assumption. Even more, depending on whether cell death occurs randomly, mimicking a cell cycle-non-specific drug or at mitosis, different selection forces act on phenotypes, which can either favor faster or slower phenotypes with drastic consequences in recurrent dynamics and therapy efficacy. In untreated populations, phenotypes with proliferative advantage thrive, as expected. Yet, fundamental differences are observed in the selection forces that act on heterogeneous populations when mitotic and random death probabilities are applied. The reason for these tremendous differences is owed to the fact that when random death probability is applied, all phenotypes in a population experience a fixed death rate. Thus, faster phenotypes will be affected; however, their increased proliferative capacity will compensate the loss of death. But slower proliferating cells will be affected the most by this probability. On the contrary, when mitotic death probability is applied, the death rate is adjusted according to the proliferation rate of each phenotype. Thus, in that case, divergent selection forces act on heterogeneous populations, favoring either faster or slower phenotypes, depending on the balance between proliferation and death processes.

We should note that the current work is theoretic and oversimplified. The work ignores the pharmacokinetics, the spatial distribution of nutrients and drugs that is developed in dense configurations, the phenotypic drift and clonal evolutionary dynamics that may alter the proliferative capacity of the daughter cells relative to their parents, the microenvironmental heterogeneity, as well as the variety of microenvironmental dependences and adaptation that may alter the phenotypes. Furthermore, the work also focuses on tumor growth driven solely by cellular proliferation, whereas migration and invasion mechanisms could be added in the future, as well as additional resistant mechanisms and trade-offs (11), more complex evolutionary mechanisms (21), a realistic 3D setting (26), and the exploration of more sophisticated therapeutic schemes, like adaptive therapy (11). We have intentionally chosen a simple starting point in order to better demonstrate the impact of heterogeneity with respect to cell cycle duration before, during, and after treatment. The aim was to gain a better insight regarding differences between heterogeneous and homogeneous populations, while distinguishing mitotic from random death rates, by investigating the role of each in cancer populations.

In our work, we have assumed that during treatment, cancer cells may die with a given probability. This probability can be ideally derived from biological experiments that quantify drug-induced cancer cell death at a single-cell level. This probability is also associated with the dose of an anticancer drug and reflects drug pharmacodynamics. When two drugs are combined consecutively (sequential chemotherapeutic scheme), the probability associated with each drug can be applied in a straightforward manner and the effect of population heterogeneity can be easily explored. When different drugs are combined simultaneously at the same time interval, then aspects from the probability theory should be considered. As an example, a recent work of Comandante-Lou et al. (27) shows how these probabilities can be combined for the case where the two different drugs act independently.

We argue that it is highly critical to identify the heterogeneity within a tumor and account for this heterogeneity when planning a therapeutic strategy. This work shows that during, as well as after/before treatment, strong selection forces act on a heterogeneous tumor population driving its post-treatment dynamics and determining the emergent resistance, which comprises one of the major reasons for therapy failure. Thus, before incorporating more complex biology and trade-offs in the mathematical modeling that inevitably exists in cancer (15), a better understanding of these principles is important. By exploiting the underlying selection forces, we could potentially delay recurrence and control tumor. The method developed here provides a framework for predicting the selection forces acting on a heterogeneous cancer population, which may lead to models that account for these evolution forces and optimize the therapeutic schemes for a specific drug compound, respectively. Based on this framework, the implications of combination therapy with multiple drugs, where each drug compound affects differently the cancer population, would be also interesting to explore and optimize accordingly in order to better control tumor growth.

## Data Availability Statement

All datasets generated for this study are included in the article/Supplementary Material.

## Author Contributions

ET conceived and designed the experiments. GT conducted the experiments. ET, GT, and VS analyzed and interpreted the data. All authors prepared and reviewed the manuscript.

## Conflict of Interest

The authors declare that the research was conducted in the absence of any commercial or financial relationships that could be construed as a potential conflict of interest.
