# Supplementary material for: Modeling How Heterogeneity in Cell Cycle Length Affects Cancer Cell Growth Dynamics in Response to Treatment
Source: Front Oncol. 2020 Sep 10;10:1552. doi: 10.3389/fonc.2020.01552 (PMC7518087; doi:10.3389/fonc.2020.01552)
Supplement: Supplementary file 5 [file Data_Sheet_1.pdf]

Supplementary Material

1 Model Parameters

Utilizing a cellular automaton mathematical model, we investigate the spatiotemporal evolution of cells, as well as the evolution of the distribution of their proliferation times, as we vary the probability of a cell to die. Differences between homogeneous and heterogeneous populations are explored in these settings, as well as differences between mitotic and random death probabilities.

We assume that tumor cells lie on a two-dimensional regular lattice. Each lattice site (20μm x 20μm) can accommodate only one tumor cell. The cells are seeded with two different initial configurations; one randomly scattered of low cell density (1%) that mimics 2D in vitro experiments and another highly compact (80%) that mimics a central plane of a 3D tumor. In the low cell density experiments the computational domain is equal to 2.5cm, whereas for the dense configuration it is equal to 0.5cm. The heterogeneity imposed regards phenotypes with different cell-cycle duration. We assume two different initial distributions for the doubling times; normal and uniform with the same mean τ and variance $\tau/5$. We assume τ equals to 24h. We started with 500 phenotypes randomly drawn from these distributions. Details of the experimental setup are summarized in Table S1 and Table S2. Each experiment has been repeated five times, in both low and highly dense initial configurations.

Various therapeutic schemes have been tested including constant, switch-on/switch-off and periodic switch-on/switch-off treatments. We have also tested a scheme where we allow cells to grow untreated for 10 days, before treatment is applied in order to resemble tumors at later growth phase. In that case, the strong selection forces have already shaped the heterogeneous populations, in which faster phenotypes have dominated in the population.

Table 1

Parameter	Sparse experiments	Dense experiments
Domain size	2.5cm	0.5cm
Tumor Radius	8mm	0.4mm
Initial density	1%	80%
Seeding population	~5000 cells	~1000 cells

Table 2

Parameter	Normal Distribution	Uniform distribution
Number of phenotypes	500	500
Mean doubling time, τ	24h	24h
Standard deviation	$\tau/5$	$\tau/5$
Range	-	[15.7, 32.3]h

2 Mitotic death in sparse initial configuration under constant treatment

The mean and standard deviation of the growth curves, as well as the evolution of the doubling times across five experiments (mean across experiments of the mean within each experiment doubling time) are illustrated in Figure S1 and Figure S2, for initially sparse configuration, when mitotic death is applied throughout the whole experiment (constant treatment). Figure S1 shows the simulations for mitotic death probabilities less than 0.5, where selection favors the faster phenotypes, whereas Figure S2 shows the results for higher mitotic death probabilities, in which selection favors the slower phenotypes. For mitotic death probability exactly equal to 0.5, no selection force is observed.

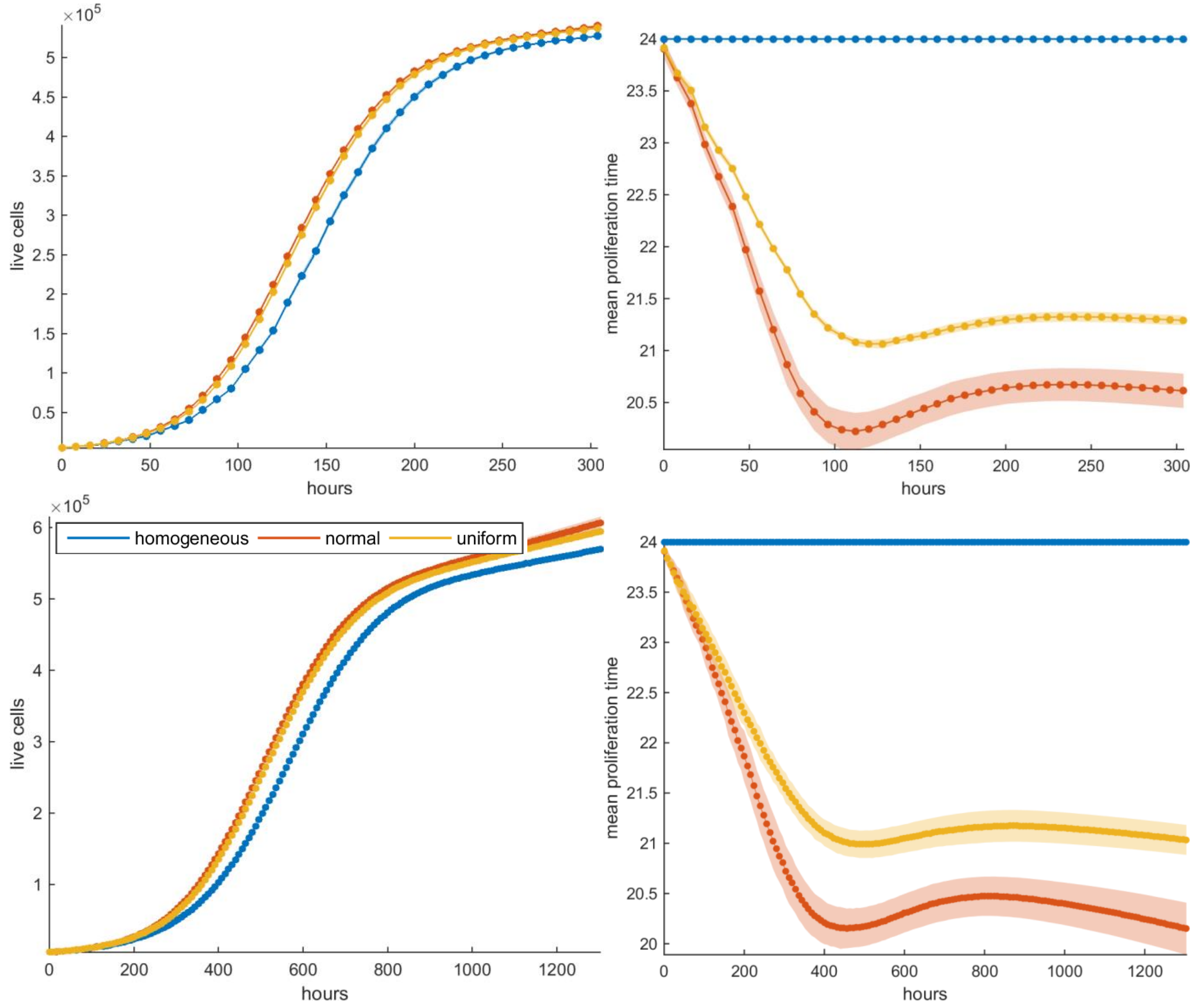

Supplementary Figure S1 Mean Growth curves (left) and mean evolution of doubling time (right) for five experiments in low initial cell density. The patches indicate the corresponding std values. Both untreated (top) and treated population with mitotic probability equal to 0.4 (bottom) are shown. Only slight variability across the experiments is observed.

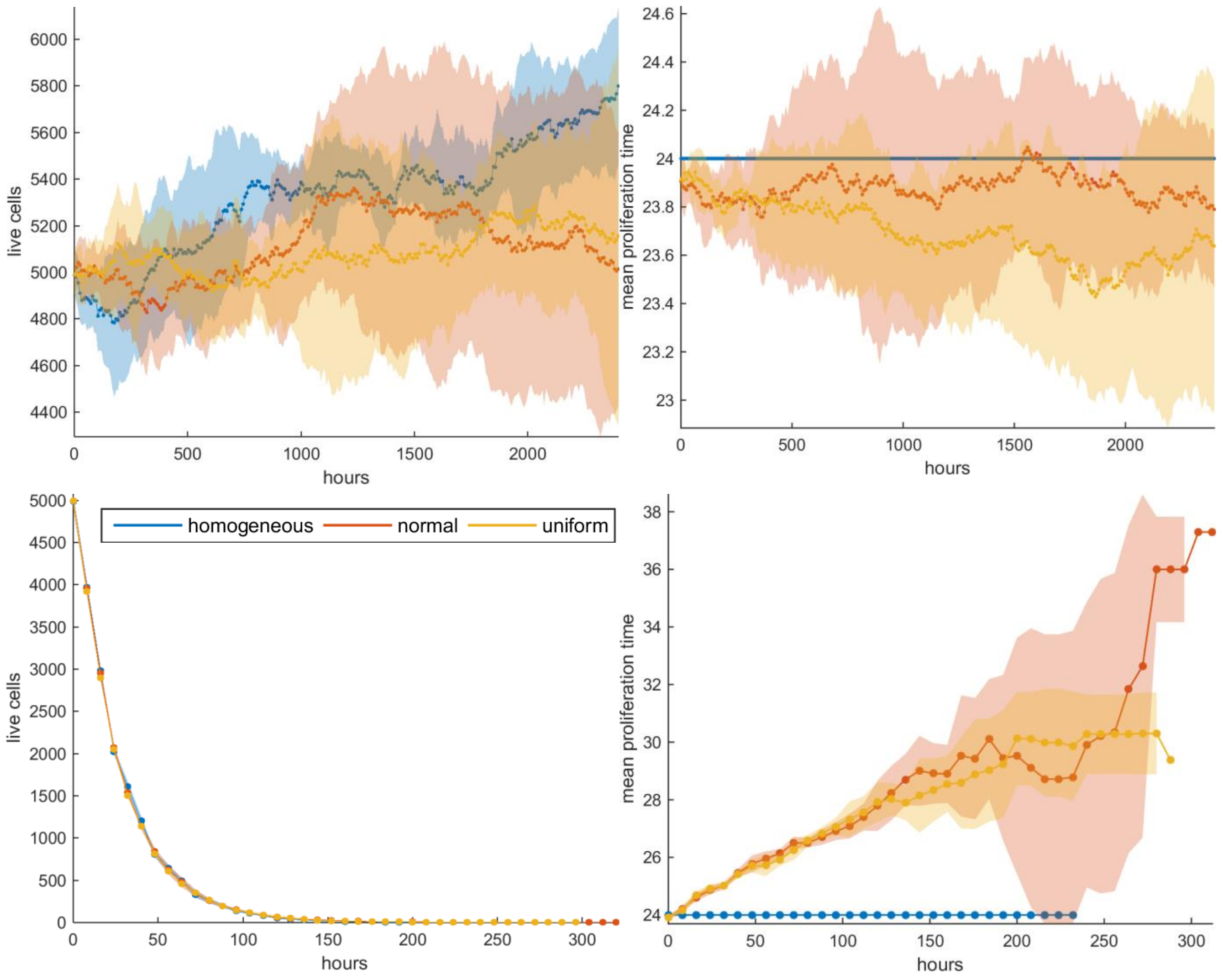

Supplementary Figure S2 Mean Growth curves (left) and mean evolution of doubling time (right) for five experiments in low initial cell density. The patches indicate the corresponding std values across the experiments. Treated population with mitotic death probability equal to 0.5 (top) and 0.8 (bottom) are shown. Due to low cell number, increased variability is observed across the experiments.

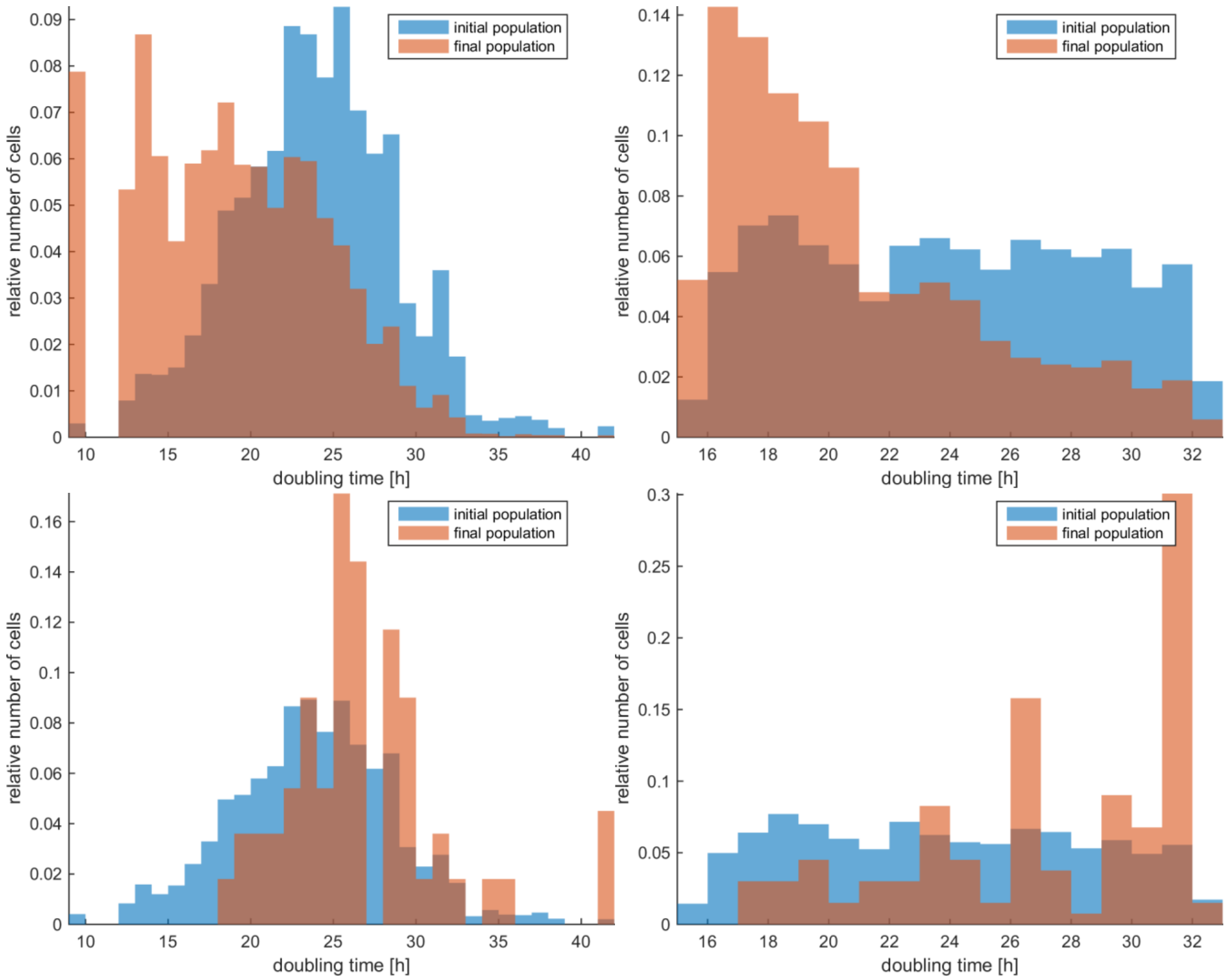

Supplementary Figure S3 Histograms of initial and final time points showing the distributions of the doubling times of the live cells. The top (bottom) row corresponds to a single experiment with mitotic death probability equal to 0.4 (0.8). The time point for the low probability is the final while for the low probability we show as final population the t= 100 hours because the final population count is zero.

3 Mitotic death in dense initial configuration under constant treatment

The mean and standard deviation of the growth curves, as well as the evolution of the doubling times across five experiments are illustrated in Figure S3 and Figure S4, for initially dense configuration, when mitotic death is applied throughout the whole experiment (constant treatment). Figure S3 shows the simulations for mitotic death probabilities less than 0.5, whereas Figure S4 shows the results for higher mitotic death probabilities.

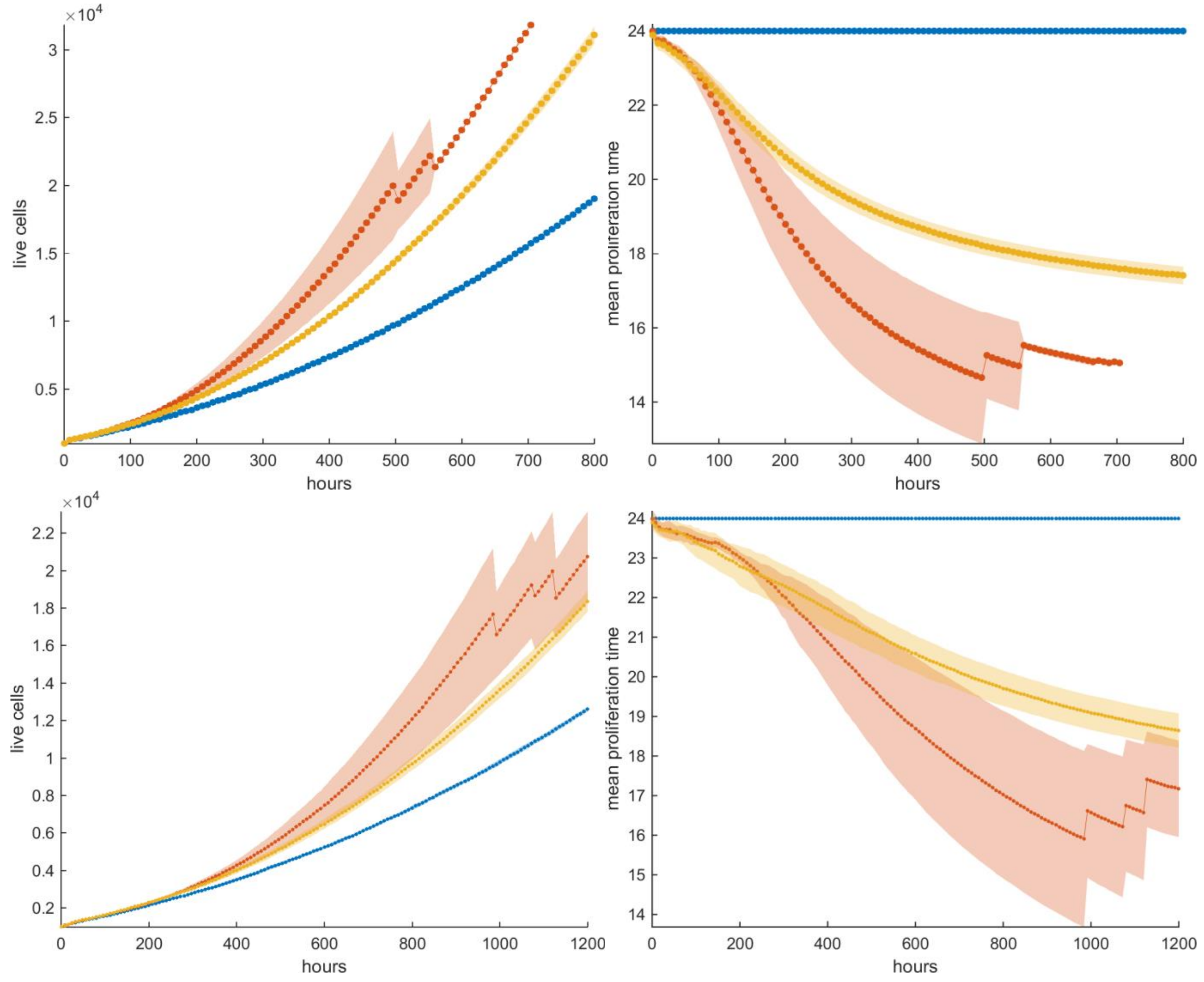

Supplementary Figure S4 Mean Growth curves (left) and mean evolution of doubling time (right) for five experiments in low initial cell density. The patches indicate the corresponding std values. Both untreated (top) and treated population with mitotic probability equal to 0.4 (bottom) are shown. Increased variability across the experiments is observed particularly for the initially normally distributed doubling times.

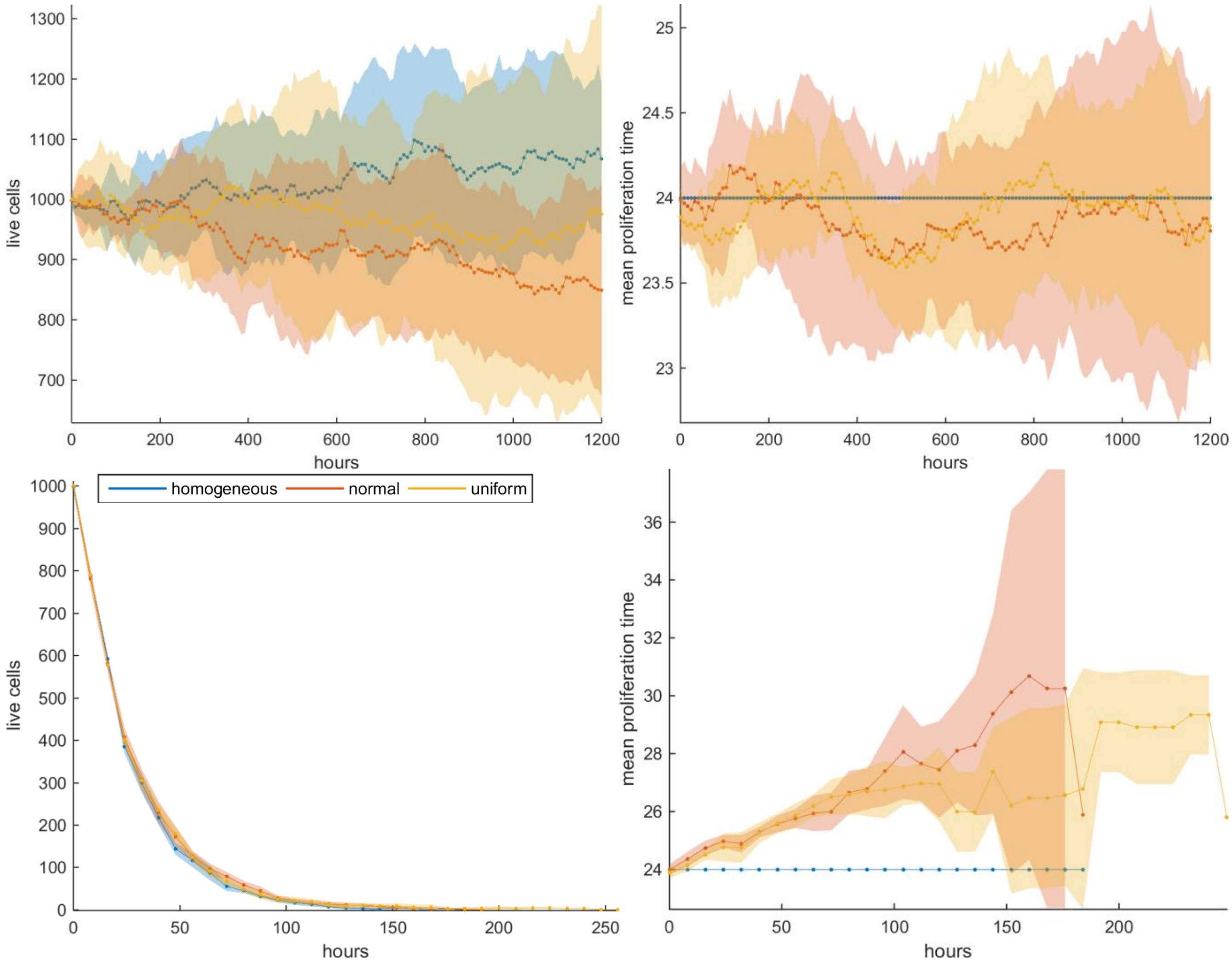

Supplementary Figure S5 Mean Growth curves (left) and mean evolution of doubling time (right) for five experiments in low initial cell density. The patches indicate the corresponding std values. Treated population with mitotic death probability equal to 0.5 (top) and 0.8 (bottom) are shown. Due to low cell number, increased variability is observed across the experiments.

4 Random death in sparse initial configuration under constant treatment

Now, death is applied randomly irrespective of the cell-cycle phase, even if the cell is quiescent. The mean and standard deviation of the growth curves, as well as the evolution of the doubling times across five experiments are illustrated in Figure S5, for initially sparse configuration, when random death is applied throughout the whole experiment (constant treatment). Random death consistently favors the faster phenotypes irrespective of the value of the probability. At high death rates (Figure S5 bottom), increased variability is observed across the experiments, due to low cell number. Few highly proliferative cells that survived treatment allow the population to regrow, driving the recurrence dynamics. High variability is observed in the recurrence dynamics.

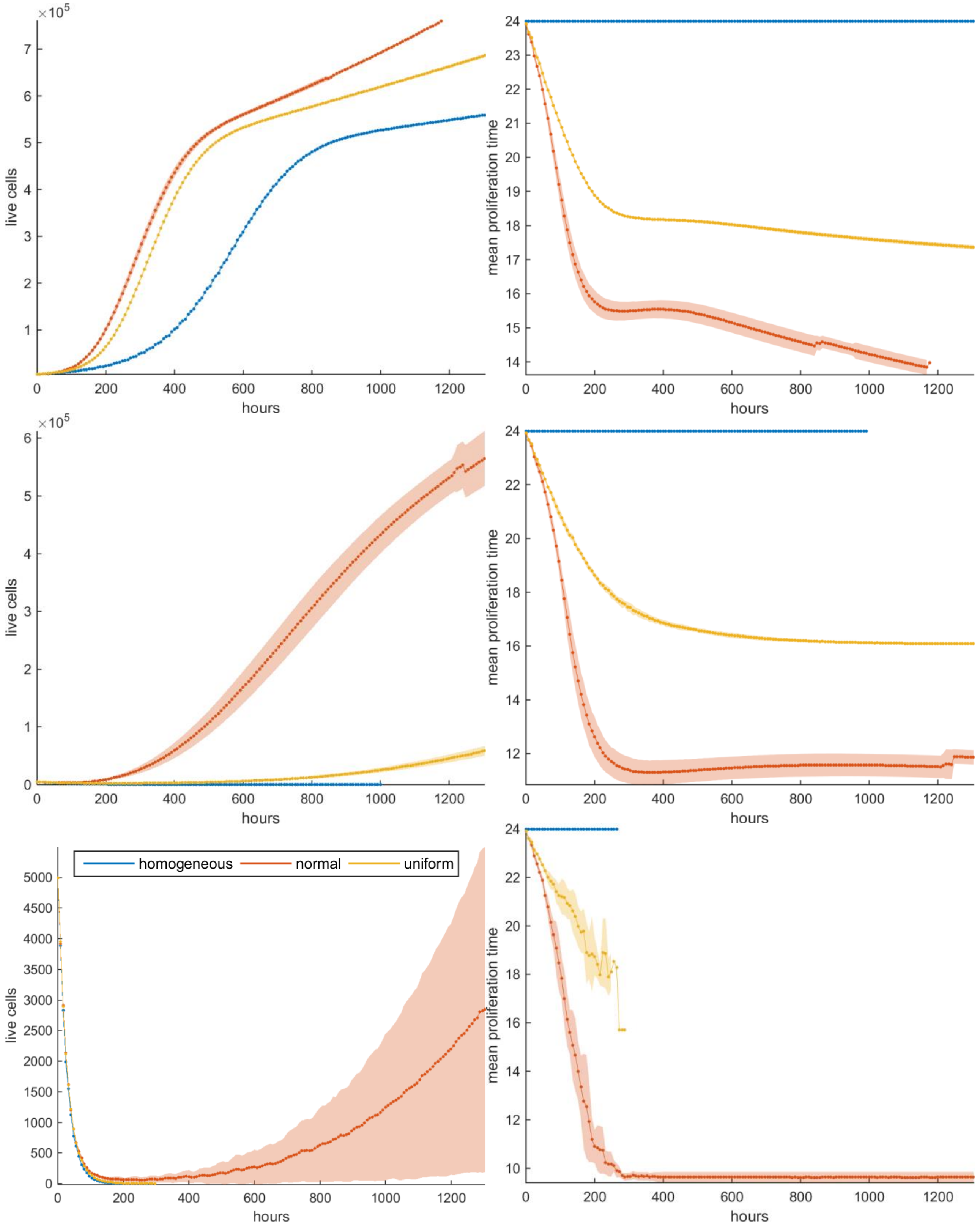

Supplementary Figure S6 Mean growth curves and the mean evolution of doubling time across five experiments. The experiments were performed in low initial cell density for random death set to equivalent to 0.4 (top), 0.6 (center) and 0.8 (bottom).

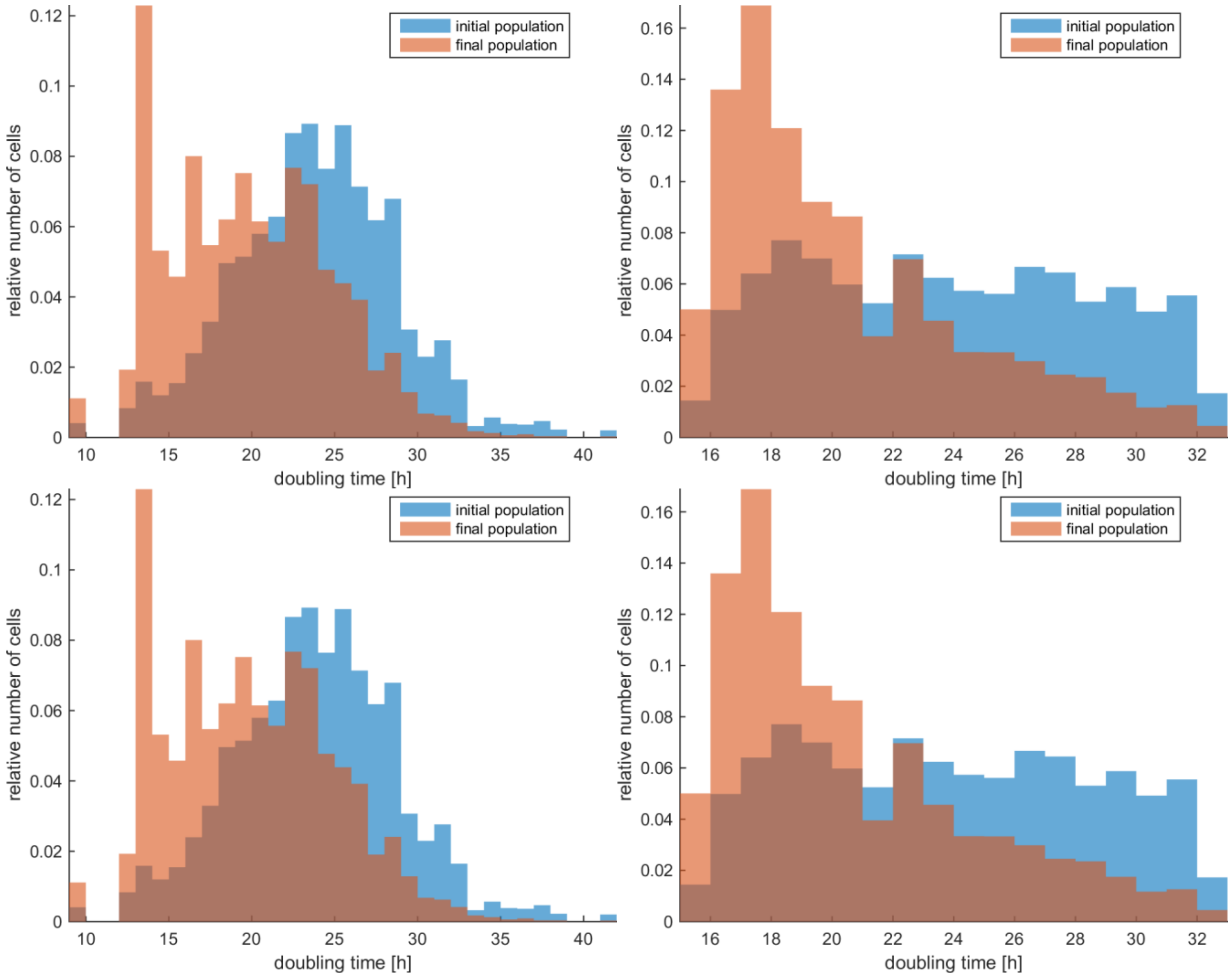

Supplementary Figure S7 Histograms of initial and final time points showing the distributions of the doubling times of the live cells. The top (bottom) row corresponds to a single experiment with random death probability equal to 0.4 (0.8). The time point for the low probability is the final while for the low probability we show as final population the t= 100 hours because the final population count is zero.

5 Random death in dense configurations compared with mitotic death under constant treatment

The mean and standard deviation of the growth curves, as well as the evolution of the doubling times across five experiments are illustrated in Figure S6, for initially dense configuration, when random death is applied throughout the whole experiment (constant treatment). The results of the mitotic death probabilities are also shown to make comparison easier.

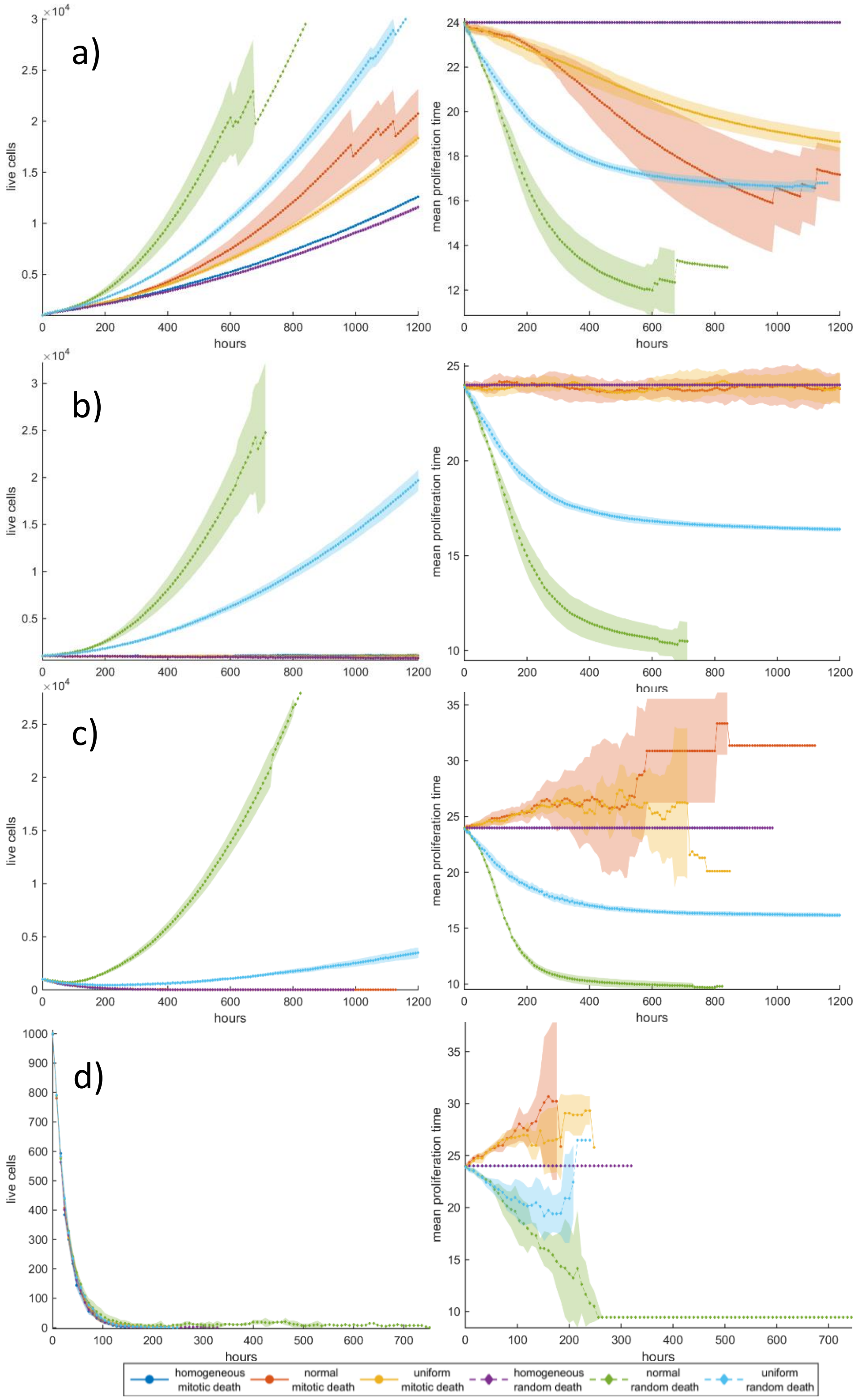

Supplementary Figure S8 Mean growth curves and the mean evolution of doubling time across five experiments. The experiments were performed in dense initial configuration. The simulations from

both mitotic and random death probabilities are illustrated for direct comparison. a) $p_m = p_r = 0.4$, b) $p_m = p_r = 0.5$, c) $p_m = p_r = 0.6$,and d) $p_m = p_r = 0.8$. Divergent selection forces are revealed.

6 Switch-on/switch-off treatment in dense configuration

We apply treatment only the first 5 days (120h) and then, we leave the population untreated. An initially highly dense configuration is assumed. The homogeneous populations have very similar dynamics before and after treatment with no differences between random and mitotic death rates. Curve fitting of the growth dynamics with second degree polynomial shows that after that re-initialization period, where the foci of surviving cells are reunited to form a new tumor, the growth dynamics of the homogeneous tumors coincide (Figure S7). Furthermore, because of the divergent selection forces that act on the tumor population during and after treatment, when high mitotic death rates are applied, the recurrence dynamics between the heterogeneous and the homogeneous population are very similar (Figure S7). On the other hand, regrowth is faster when random death rates are applied compared to mitotic rates (note the coefficient of the second-degree term). Curve fitting of the untreated population is also shown for comparison (Figure S8). We observe that the selection of slower phenotypes during treatment at high mitotic death rates results in slower recurrent dynamics relative to the untreated population, whereas when random death is applied the recurrent dynamics are very similar with the untreated case.

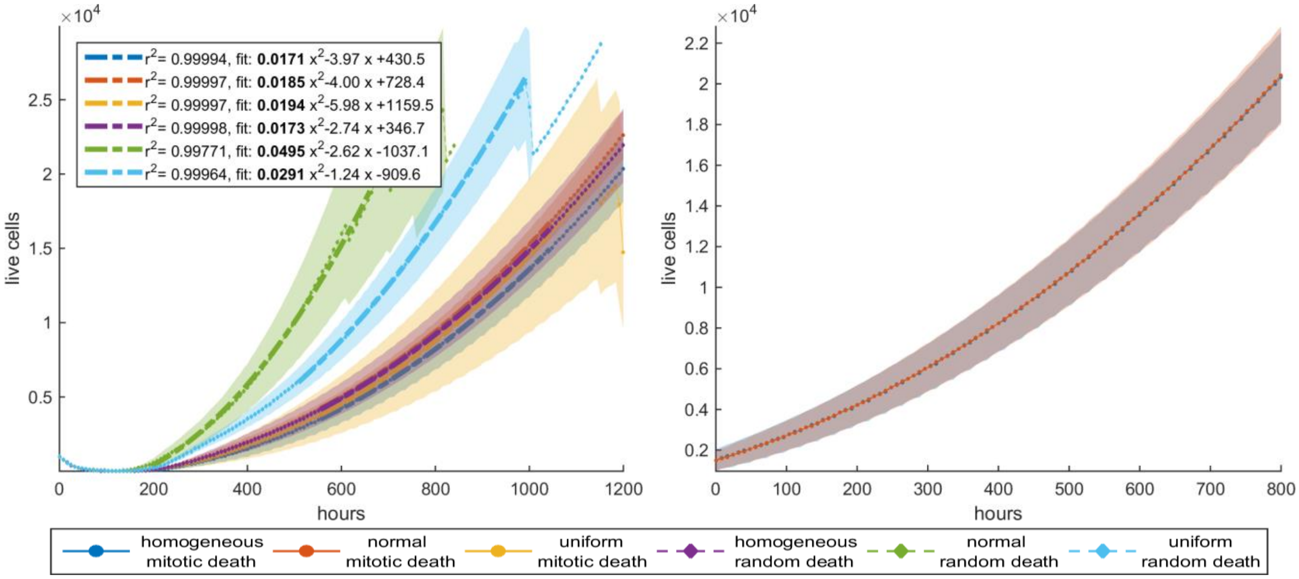

Supplementary Figure S9 Mean growth curves across five experiments. Treatment is applied for the first 5 days and then the population is left untreated. The experiments were performed in dense initial configuration. (Left) The simulations from both mitotic and random death probabilities are illustrated for direct comparison. (Right) The homogeneous curves were shifted for illustration purposes in

order to show that after that re-initialization period, the growth dynamics of the homogeneous tumors under high random and mitotic death rates coincide.

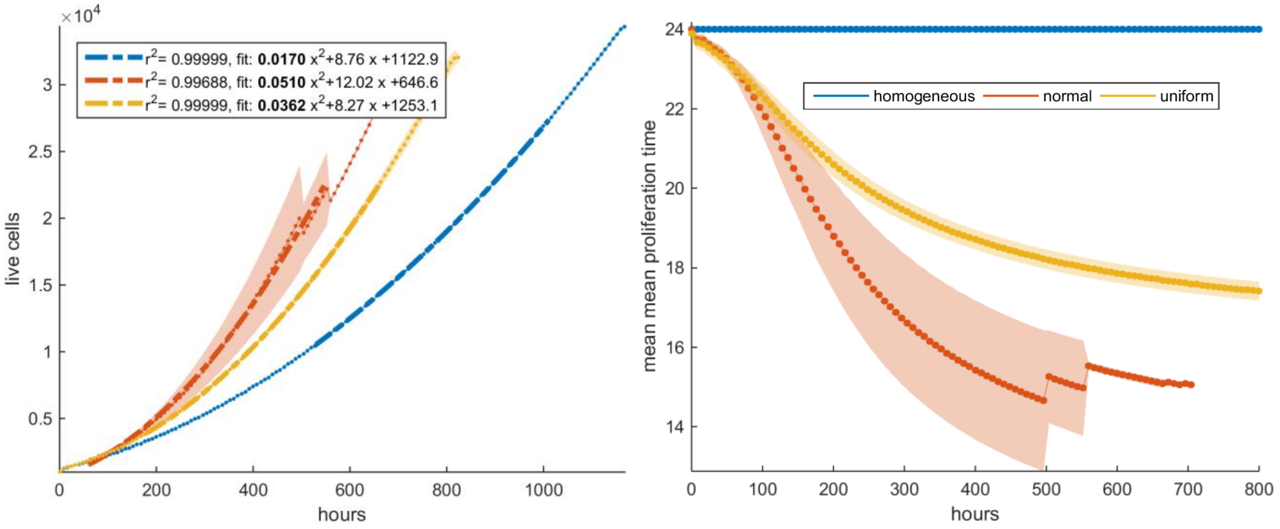

Supplementary Figure S10 Mean growth curves (Left) and the mean evolution of doubling time (Right) across five experiments. The evolution of the untreated population is shown when the initial distribution of phenotypes is homogeneous (blue line), heterogeneous derived from normal distribution (red line), and heterogeneous but derived from uniform distribution. The experiments were performed in dense initial configuration. Curve fitting of the untreated population is also shown for comparison with the treated populations.

7 Later phase growth dynamics

Considering that strong selection forces acting on the heterogeneous populations, we tested also the case where we allow cells to grow untreated for 10 days, before treatment is applied in order to resemble tumors at later growth phase. As we have previously described, during the untreated period, selection of the faster phenotypes has occurred, and the distribution of the proliferation time has been right-skewed. With this initialization, treatment is then applied. We observed similar selection forces acting on the heterogeneous population, before and after treatment, further highlighting that heterogeneity and the range of this heterogeneity is that plays a major role in treatment and post-treatment dynamics and not so the abundance of each phenotype in the population.

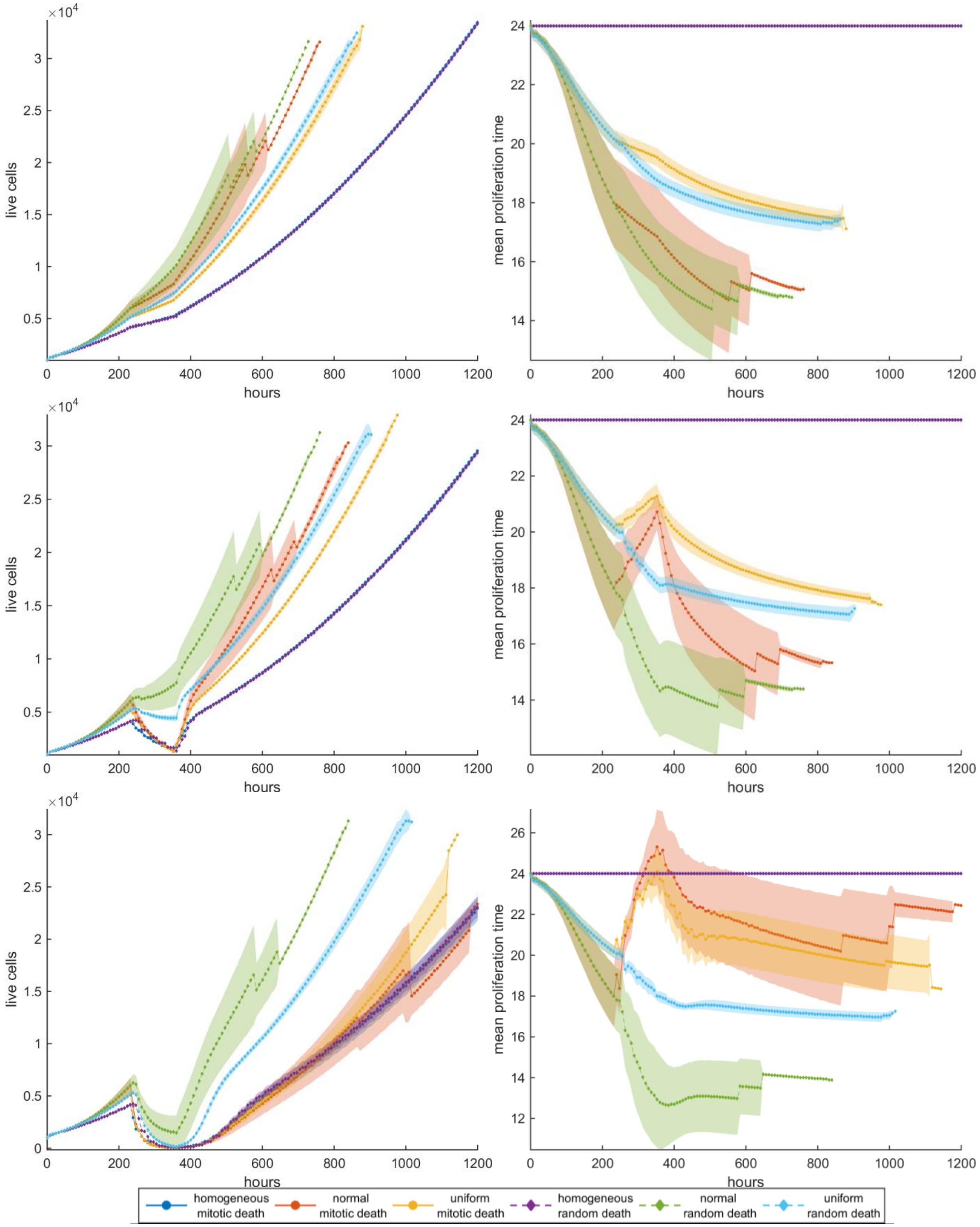

Supplementary Figure S11 Mean growth curves and the mean evolution of doubling time across five experiments. The experiments were performed in dense initial configuration. The simulations from both mitotic and random death probabilities are illustrated for direct comparison. The mitotic death probability and the equivalent random were set to equivalent to 0.4 (top), 0.6 (center) and 0.8 (bottom).
